# An improved inverse-type Ca^2+^ indicator can detect putative neuronal inhibition in *Caenorhabditis elegans* by increasing signal intensity upon Ca^2+^ decrease

**DOI:** 10.1371/journal.pone.0194707

**Published:** 2018-04-25

**Authors:** Sayuri Hara-Kuge, Tomonobu Nishihara, Tomoki Matsuda, Tomohiro Kitazono, Takayuki Teramoto, Takeharu Nagai, Takeshi Ishihara

**Affiliations:** 1 Department of Biology, Faculty of Science, Kyushu University, Fukuoka, Japan; 2 Core Research for Evolutional Science and Technology (CREST), Japan Science and Technology Agency, Saitama, Japan; 3 Department of Biomolecular Science and Engineering, The Institute of Scientific and Industrial Research, Osaka University, Osaka, Japan; 4 Graduate School of Systems Life Sciences, Kyushu University, Fukuoka, Japan; Cinvestav-IPN, MEXICO

## Abstract

Sensory processing is regulated by the coordinated excitation and inhibition of neurons in neuronal circuits. The analysis of neuronal activities has greatly benefited from the recent development of genetically encoded Ca^2+^ indicators (GECIs). These molecules change their fluorescence intensities or colours in response to changing levels of Ca^2+^ and can, therefore, be used to sensitively monitor intracellular Ca^2+^ concentration, which enables the detection of neuronal excitation, including action potentials. These GECIs were developed to monitor increases in Ca^2+^ concentration; therefore, neuronal inhibition cannot be sensitively detected by these GECIs. To overcome this difficulty, we hypothesised that an inverse-type of GECI, whose fluorescence intensity increases as Ca^2+^ levels decrease, could sensitively monitor reducing intracellular Ca^2+^ concentrations. We, therefore, developed a Ca^2+^ indicator named inverse-pericam 2.0 (IP2.0) whose fluorescent intensity decreases 25-fold upon Ca^2+^ binding *in vitro*. Using IP2.0, we successfully detected putative neuronal inhibition by monitoring the decrease in intracellular Ca^2+^ concentration in AWC^ON^ and ASEL neurons in *Caenorhabditis elegans*. Therefore, IP2.0 is a useful tool for studying neuronal inhibition and for the detailed analysis of neuronal activities *in vivo*.

## Introduction

In the central nervous system, sensory information is co-ordinately processed by excitatory and inhibitory neuronal activities. These neuronal activities have been studied by electrophysiology [1, 2] and live imaging using fluorescent chemicals [3–6] and genetically encoded Ca^2+^ indicators (GECIs) [7–12]. GECIs, including FRET (Förster Resonance Energy Transfer)-based indicators such as Cameleons [13–15] and single-fluorophore indicators, such as the GCaMP family, make it possible to analyse neuronal activities of specific neurons *in vivo* by using gene promoters that express the proteins in specific types of neuron [16–18]. In addition, recent improvements to GCaMPs enables single action potentials in living animals to be detected, and red fluorescent Ca^2+^ indicators, such as R-GECO and RCaMPs, have been also developed [19–23]. These GECIs are sufficiently sensitive to increases in Ca^2+^ concentration that neuronal excitation can easily be detected. On the other hand, most GECIs have difficulty in detecting decreases in Ca^2+^ concentration from the resting phase, because they have been optimized to monitor increases in Ca^2+^ concentration. For example, the fluorescence of GCaMPs under the resting phase is very dim and thereby the signal to noise ratio is low. Therefore, Ca^2+^ concentration lower than that at the resting phase cannot be reliably detected. This is mainly because the *K*_d_ values of most GECIs do not correspond to lower Ca^2+^ concentrations. Moreover, a decrease in fluorescence intensity can incidentally occur with a change of intracellular conditions, such as pH [24]; therefore, a decrease in fluorescence is not always caused by a decrease in Ca^2+^ concentration. Therefore, in addition to the ordinary GECIs, a new type of GECI that can monitor decreases in Ca^2+^ concentration from the resting phase is needed to investigate neuronal function because it would reflect neuronal inhibition. The development of sensitive GECIs that detect decreases in Ca^2+^ concentration may lead to a new chapter of Ca^2+^ imaging studies for the investigation of neuronal processing *in vivo*.

We previously reported various “pericam” types of indicators that are based on circularly permuted yellow fluorescent protein (cpYFP) fused to calmodulin and calmodulin-target peptide, M13 [8]. Among these, “inverse-pericam” has a unique property of being a quenching type Ca^2+^ indicator; its fluorescence intensity becomes 7-fold dimmer upon Ca^2+^ binding [8]. This inverse-pericam and another inverse-type Ca^2+^ indicator, Y-GECO1 have been already used for Ca^2+^ imaging to detect Ca^2+^ change in brain slices [25, 26]. But these indicators have not been used to monitor the decrease of Ca^2+^ concentration in neurons of living animals.

Here we report an improved inverse pericam 2.0 (IP2.0), generated by mutagenesis of the original inverse-pericam. IP2.0 can sensitively monitor Ca^2+^ oscillation in HeLa cells. Furthermore, by expressing IP2.0 in *Caenorhabditis elegans*, we succeeded in monitoring both decreases and increases in Ca^2+^ concentration in the chemosensory neurons, AWC^ON^ and ASEL. These responses may represent inhibition and excitation of these sensory neurons, respectively. Therefore, IP2.0 is useful for monitoring changes in Ca^2+^ concentration *in vivo* and may enable the sensitive and simultaneous detection of neuronal excitation and inhibition in neuronal circuits *in vivo* by combined monitoring with conventional red GECIs, such as RCaMPs.

## Results

### Development of improved inverse-pericam

**Fig 1:**
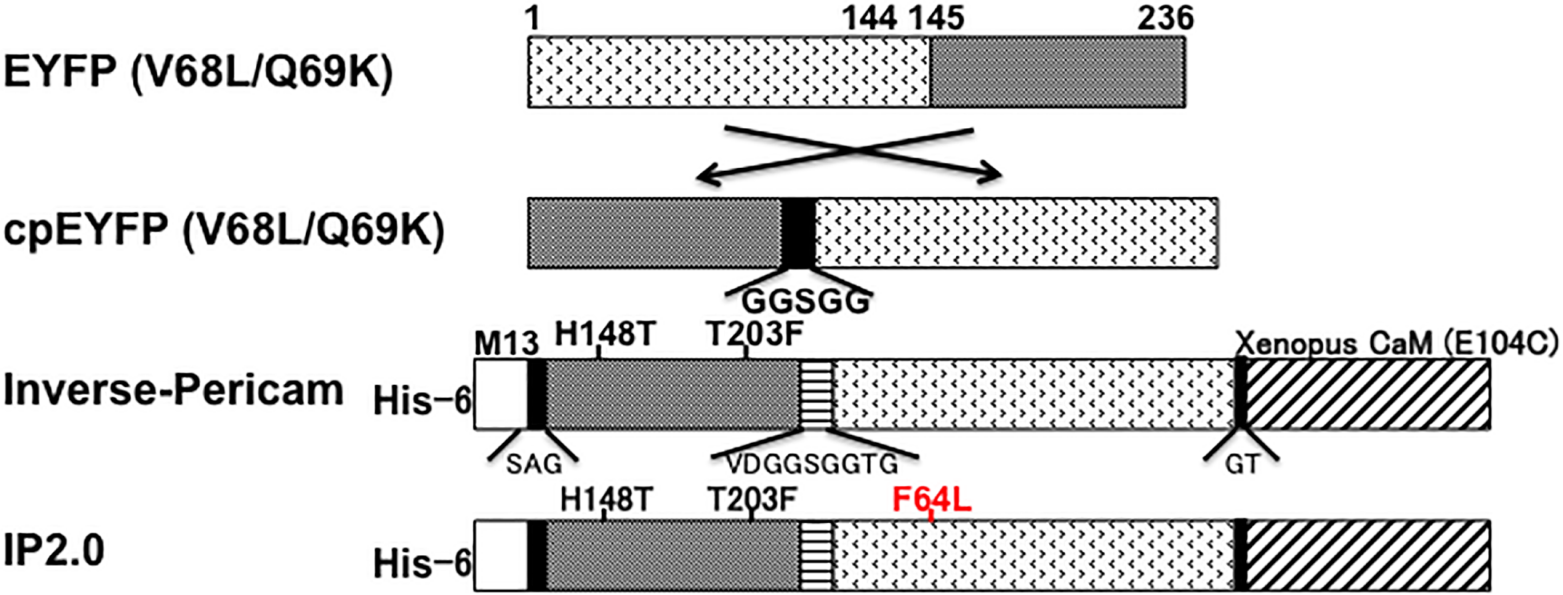
Schematic structures and sequences of inverse-pericam and IP2.0. Sequences of linkers and amino and substitutions are shown below and above the bars, respectively. His-6: the polyhistidine tag.

**Fig 2:**
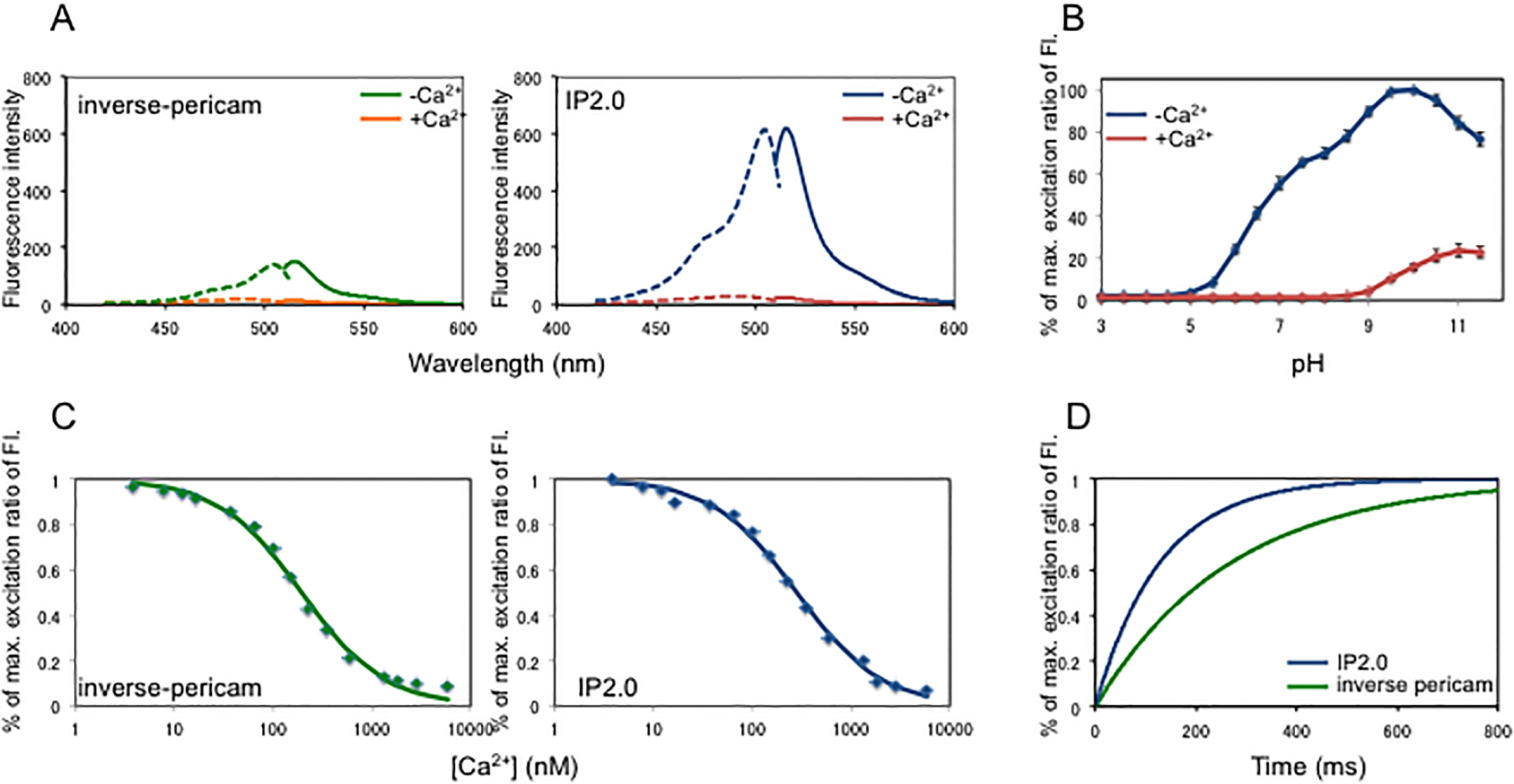
*In vitro* properties of IP2.0. **A**, Normalized fluorescence excitation (dashed lines) and emission (solid lines) spectra in Ca^2+^-free (green and blue lines) and Ca^2+^-saturated (orange and red lines) states. **B**, pH-dependency of normalized amplitudes at the 515 nm excitation peak in Ca^2+^-free (blue line) and Ca^2+^-saturated (red line) states. **C**, Ca^2+^ titration curve of inverse-pericam (left) and IP2.0 (right). **D**, Fluorescence rise response of inverse-pericam (green line) and IP2.0 (blue line) to a stepped decrease in [Ca^2+^]_free_ from 10 μM to < 10 nM. The raw data of Fig 2D is available in figshare (https://doi.org/10.6084/m9.figshare.5976067.v1).

Neuronal excitation in living animals is often analysed using GCaMPs, whose fluorescence intensities increase as intracellular Ca^2+^ concentration increases. We considered that Ca^2+^ indicators that monitor decreases in Ca^2+^ concentration through increasing fluorescence intensities could complement GCaMP-type GECIs to enable analysis of both neuronal excitation and inhibition. Therefore, we sought to improve inverse-pericam, one of the various pericams, whose fluorescence increases as Ca^2+^ concentration decreases [8]. Inverse-pericam is a GECI with a M13 peptide, cpYFP and calmodulin domain-like GCaMP. Its green fluorescence, at 500 nm excitation, decreases by 15% with Ca^2+^ [8]; however, it has not been used for the sensitive detection of intracellular Ca^2+^ concentration *in vivo*. To obtain improved inverse-type Ca^2+^ probes from inverse-pericam, we screened colonies expressing inverse-pericam mutants generated by error prone PCR (see Methods). After screening about 20,000 colonies, we obtained an improved inverse-type Ca^2+^ indicator, named IP2.0, which has only one substitution, F64L, compared with inverse-pericam (Fig 1). IP2.0 exhibited similar excitation and emission spectra to inverse-pericam [8]. IP2.0, excited at 488 nm, showed an emission peak at 514.5 nm without Ca^2+^, which is close to that of inverse pericam at 515 nm. The intensity at the fluorescence spectrum peak of IP2.0 was approximately three times higher than that of inverse-pericam in the absence of Ca^2+^ (Fig 2A and Table 1) and the dynamic range at 515 nm between the presence and absence of Ca^2+^ was approximately two times larger for IP2.0 than for inverse-pericam (Fig 2A, Table 1 and S1 File). Indeed, IP2.0 excited for the optimal emission peak showed 25-fold greater fluorescence at 520 nm without Ca^2+^ compared to that with Ca^2+^ (Fig 2A). Both Ca^2+^-bound and Ca^2+^-free IP2.0 were pH-titrated in a similar way and the Ca^2+^-free protein was about 7-fold brighter than the Ca^2+^-bound form in the ionized state (pH>8.0) (Fig 2B and S2 File) [8]. In the neutral pH region, the fluorescence of IP2.0 appeared bright enough for *in vivo* studies. From the Ca^2+^ titration curve, the *K*_d_ value of IP2.0 could be calculated as 284 nM (Fig 2C and S3 File) and it was lower than that of inverse-pericam (Table 1) and slightly higher than that of GCaMP3 [17, 18, 27], or GCaMP6f [18]. Ca^2+^-binding to inverse-pericam and IP2.0 was accompanied with Hill coefficients close to 1.0, similar to values shown for GCaMP families [18, 21]. To measure the response kinetics of inverse-pericam and IP2.0, we used stopped-flow fluorometry. In a stepped reduction of free Ca^2+^ concentration from 10 μM to zero (<10 nM), both inverse-pericam and IP2.0 responded with a double exponential time course (Fig 2D). The dissociation constant *k*_off_ was calculated as 280 ms^-1^ for inverse-pericam and as 128 ms^-1^ for IP2.0 (Table 1) and these responses were similar to those of GCaMP variants [17, 18]. These results indicate that IP2.0 is a good candidate for the measurement of intracellular Ca^2+^ *in vivo*, especially for decreases in Ca^2+^ concentration.

**Table 1:** Spectral characteristics of inverse-pericam and IP2.0.

Variant	Mutation^a^	*K*_d_ for Ca^2+^ (*n*)^b^	Relative *F*_*max*_	Dynamic Range (*F*_max_*-F*_min_*/F*_min_)	Rise *t*_1/2_
Inverse-pericam	H148T T203F	196 nM (1.03)	1.00	6.7	280 msec
IP2.0	H148T T203F F64L	284 nM (1.02)	2.95	25.0	128 msec

^a^Substitutions from primary sequence of EYFP (V68L/Q69L) are given as the single-letter code for the amino acid being replace, its numerical position in the sequence, and the single-letter code for replacement.

^b^*K*_d_ values for Ca^2+^ were measured from fitted curves in Fig 2C. *n* in parentheses is the Hill coefficient.

### Imaging in HeLa cells expressing inverse-pericam and IP2.0

**Fig 3:**
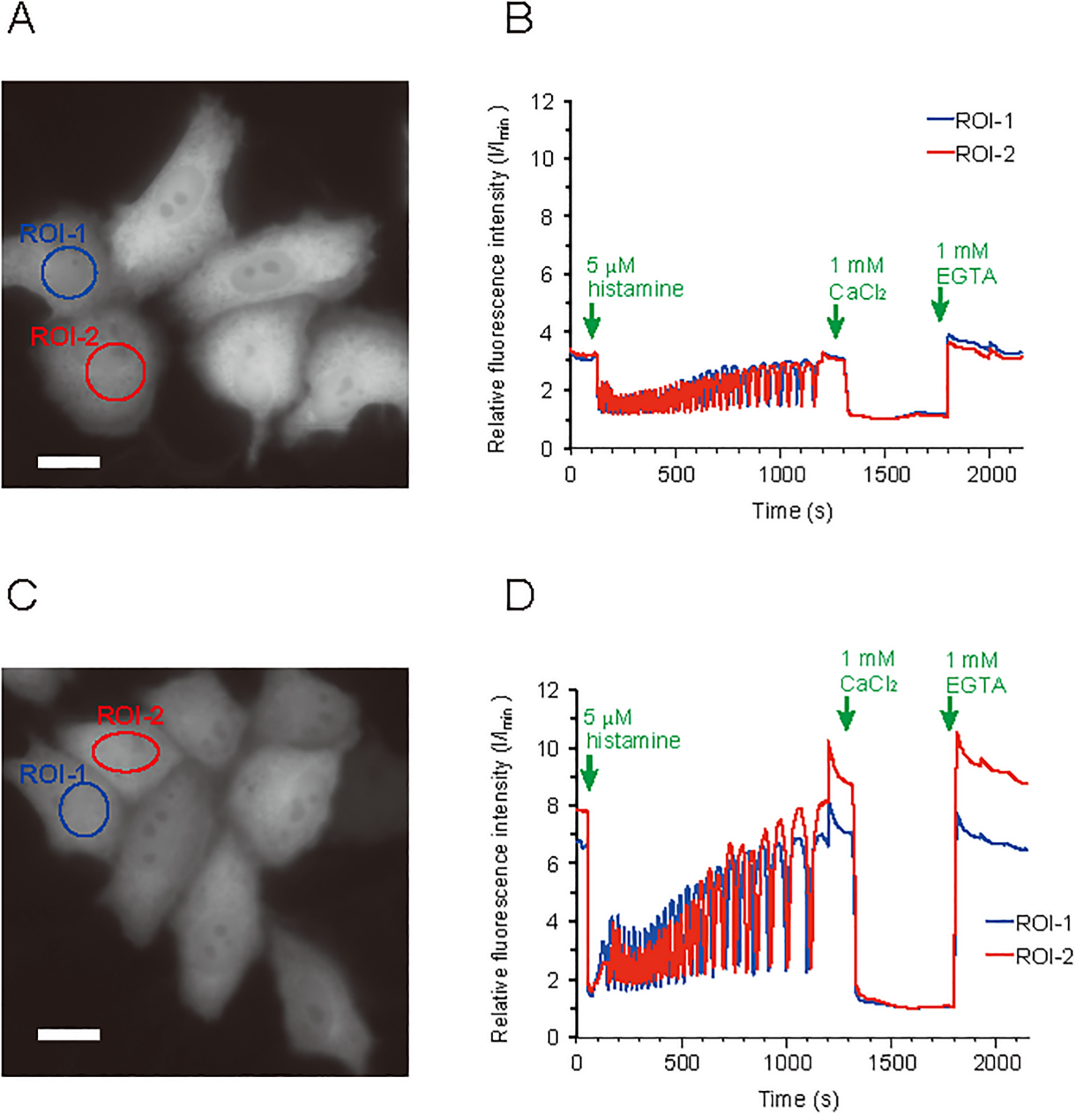
Representative Ca^2+^ imaging in HeLa cells. Fluorescence images of HeLa cells (**A, C**) and fluorescence intensity vs. time traces (**B, D**) in the ROIs of fluorescence images. Images were taken of HeLa cells transfected with inverse-pericam (**A, B**) and IP2.0 (**C, D**). Scale bar: 20 μm. The raw data of Fig 3B and D is available in figshare (https://doi.org/10.6084/m9.figshare.5976610.v1).

We examined whether IP2.0 could be used to analyse intracellular Ca^2+^ concentration using HeLa cells. We transfected cDNA for inverse-pericam or IP2.0 into HeLa cells and monitored the change of intracellular free Ca^2+^ concentration after histamine stimulation. Stimulation of HeLa cells by histamine induces Ca^2+^ oscillations; therefore, the various GECIs have been tested in HeLa cells [8, 19]. Similarly to inverse-pericam, we observed oscillations in the fluorescence intensity of IP2.0 following histamine stimulation (Fig 3). The response of IP2.0 was about three times larger than that of inverse-pericam and the changes in fluorescence seemed to be opposite to those of flash-pericam [8] or GECO variants [19]. We performed additional experiments using 44 HeLa cells expressing IP2.0. S1 Fig showed the ratios of the fluorescent intensity of the first spike after histamine stimulation to the basal intensity (S4 File). This analysis revealed that the intensity of IP2.0 consistently changed to the stimulation without being affected by the difference of the expression level among cells. From these results, we suggest that IP2.0 can be used as a practically useful inverse type of Ca^2+^ indicator in living animals.

### Imaging of Ca^2+^ concentration in *C*. *elegans* neurons

**Fig 4:**
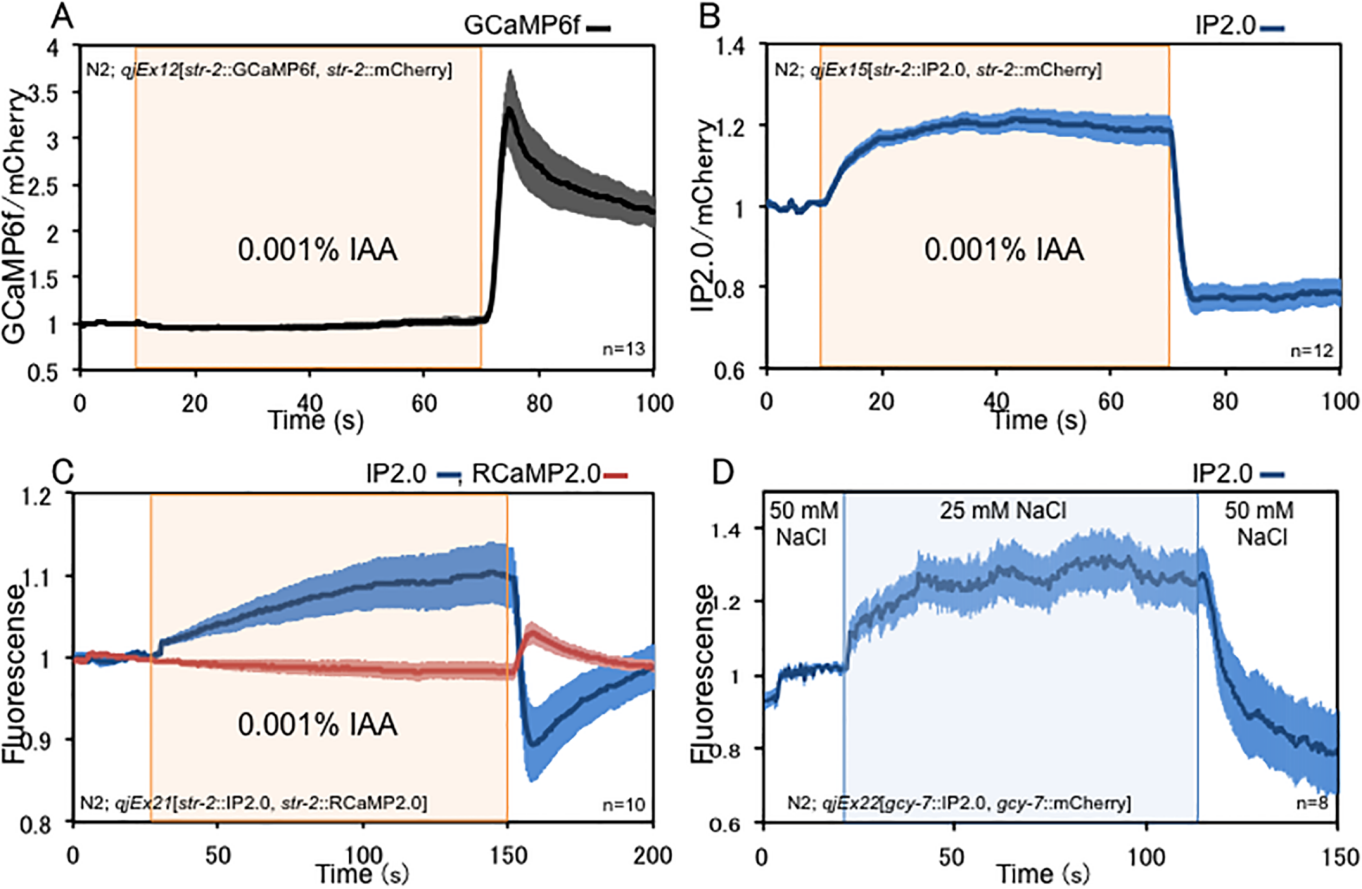
*In vivo* imaging of Ca^2+^ responses in *C*.*elegans*. (A) GCaMP6f Ca^2+^ response to isoamylalcohol in AWC^ON^ (n = 13). (B) IP2.0 Ca^2+^ response to isoamylalcohol in AWC^ON^ (n = 12). (C) Dual-colour of RCaMP2.0 and IP2.0 Ca^2+^ responses to isoamylalcohol in AWC^ON^ (n = 10). (D) IP2.0 Ca^2+^ response to change of NaCl concentration in ASEL (n = 8). The values are shown as relative to *F*_*0*_ and error bars represent SEM. The raw data of Fig 4 is available in figshare (https://doi.org/10.6084/m9.figshare.5976619.v1).

Next, we analysed neuronal activity using IP2.0 in living *C*. *elegans* to test its *in vivo* performance. We used unanaesthetised *C*. *elegans* to monitor the activity of the AWC^ON^ chemosensory neuron, in which the fluorescence of GCaMPs decreases in response to odour stimulation and increases with odour removal (S2 Fig) [28, 29]. IP2.0 or GCaMP6f [18] was expressed in AWC^ON^ neurons together with mCherry, which was used as an internal fluorescent standard to prevent motion artefacts by calculating the fluorescence ratios of IP2.0 or GCaMP6f to mCherry. Before Ca^2+^ imaging, we checked whether transgenic worms are affected by the expression of GECIs and found that worms expressing GCaMP6f or IP2.0 in AWC^ON^ exhibited normal AWC-dependent chemotaxis (S3 Fig and S4 File), suggesting that the expression of GECIs did not affect the behaviour in these strains. Since AWC^ON^ neurons expressing GCaMP respond to 0.01–0.0001% isoamylalcohol (IAA) [28, 30], individual worms were imaged in a microfluid chamber [31] during addition and after removal of 0.001% IAA, which the AWC^ON^ neuron senses. We confirmed that IP2.0 also detected neuronal activities of AWC^ON^ neurons responding to 0.01–0.0001% IAA (S4 Fig). The increase of GCaMP6f fluorescence can be observed in AWC^ON^ neurons upon IAA removal as reported using GCaMP3 [29], whereas a decrease in fluorescence upon IAA stimulation was not evident in two independent lines (Fig 4A and S5A Fig). This result is very similar to that using other GCaMPs [28–30, 32, 33] suggesting that GCaMP6f, is suitable for monitoring the increase of Ca^2+^ concentration and for analysing neuronal excitation. On the other hand, the decrease of Ca^2+^ concentration upon IAA stimulation can be clearly monitored by increased IP2.0 fluorescence (Fig 4B and S5B Fig), suggesting that IP2.0 can sensitively detect neuronal inhibition. When IP2.0 was simultaneously expressed with RCaMP2.0 [21] in the AWC^ON^ neuron, the fluorescence intensity of RCaMP2.0 was increased by removal of IAA, similar to GCaMP6f, while the fluorescence intensity of IP2.0 was decreased by removal of IAA and increased by IAA stimulation. When two different types of Ca^2+^ indicator were expressed in an AWC^ON^ neuron simultaneously, the changes of intracellular Ca^2+^ concentration seemed more reliable and easier to be understood (Fig 4C).

Next, we analysed the neuronal activities of ASEL neurons, which respond to NaCl concentration, by expressing IP2.0. The ASEL neuron is known to respond to an increase in NaCl concentration [34]; however, general calcium probe did not detect a change in Ca^2+^ concentration in response to a decrease in NaCl concentration. The fluorescent intensity of IP2.0 increased in response to a decrease in NaCl concentration, indicating that Ca^2+^ concentration in the ASEL neuron decreased in response to the decrease in NaCl concentration (Fig 4D). Next, we carried out the simultaneous measurement of the fluorescent changes of IP2.0 and RCaMP2.0 in ASEL neurons of another independent transgenic line. The fluorescent changes of RCaMP2.0 can be observed only when ASELresponded to the decrease in NaCl concentration, whereas those of IP2.0 were observed when ASEL responded to both of increase and decrease in NaCl concentration (S5C Fig). These results suggest that IP2.0, which can be used with various red fluorescent Ca^2+^ probes, is suitable for studying sensitive neuronal activities that cannot be detected by GCaMPs or RCaMPs.

## Discussion

For the last decade, GECIs, especially indicators with one fluorophore, such as GCaMPs, have been actively developed. They have found different applications in *in vivo* studies depending on their characteristics, such as colour, affinities to Ca^2+^, optimal pH or Ca^2+^ binding speeds [35–37]. Accordingly, Ca^2+^dynamics have been analysed in cultured cells and in many species *in vivo*, including, mouse, rat, *Drosophila*, zebrafish and *C*. *elegans* [11, 17–23, 29, 32, 38–44]. However, these fluorescent Ca^2+^ indicators are used mainly for detecting neuronal excitation, because their fluorescence increases when the expressing neuron is excited. To understand the informational processing in neuronal circuits, which are finely controlled by the combination of excitation and inhibition, simultaneous analysis of not only neuronal excitation but also neuronal inhibition may be helpful.

We developed an inverse-type Ca^2+^ indicator, IP2.0, from inverse-pericam. We introduced random mutations into inverse-pericam by error-prone PCR. By comparing the fluorescent intensities of colonies expressing mutagenized inverse-pericam between conditions with and without Ca^2+^, we screened about 20,000 clones and isolated IP2.0. IP2.0 becomes dimmer with increasing of Ca^2+^ concentration and brighter with decreasing of Ca^2+^ concentration, which makes it possible to monitor neuronal inhibition at least in *C*. *elegans*. IP2.0 was changed from inverse pericam by only one substitution, F64L. F64L is important for chromophore formation and for brightness, and this substitution was also introduced into EGFP, when EGFP was developed from GFP [45]. The structural study on EGFP made it clear that replacement of Phe64 with Leu in EGFP causes subtlety of the hydrophobic core packing to the chromophore and reduces surface exposure of two hydrophobic residues [46].

The *K*_d_ value of IP2.0 is 285 nM, which is close to that of other indicators, including GCaMPs and YC3.60 [15]; therefore, IP2.0 can be a good Ca^2+^ indicator for monitoring intracellular Ca^2+^ concentration.

The most unique characteristic of IP2.0 is that it is bright at low Ca^2+^ concentrations; neurons expressing IP2.0 can be easily observed even at resting state, and thereby subtle changes in fluorescence intensity can be sensitively monitored even at low Ca^2+^ concentrations. This is in contrast to most Ca^2+^ indicators, which are so dark at the resting state that it is difficult to identify the cells expressing the Ca^2+^ indicator.

IP2.0 differs from inverse-pericam in various *in vitro* characteristics (Table 1) and the change in fluorescence intensity of IP2.0 by histamine stimulation of HeLa cells was about three times larger compared with that of inverse-pericam. Our *in vivo* studies using *C*. *elegans* showed that IP2.0 is a unique GECI for the sensitive detection of neuronal inhibition. We detected not only neuronal inhibition during IAA stimulation in AWC^ON^ neurons (Fig 4A–4C) but also a change of intracellular Ca^2+^ concentration in ASEL neurons depending on a decrease in NaCl concentration (Fig 4D). Though ASEL neurons are well known to respond to an increase in NaCl concentration [34], it has not been previously reported that it also responds to a decrease in NaCl concentration. According to these results, IP2.0 may be possible to monitor the decrease of intracellular Ca^2+^ concentrations coinciding with neuronal inhibition in many other neuronal subtypes where other GECIs have been unable to detect decreasing intracellular Ca^2+^ concentrations. Moreover, IP2.0 will be useful for the large-scale recording of neuronal activities in unanaesthesia *C*. *elegans*, which will aid investigations of how populations of neurons generate animal behaviour [47–49].

IP2.0 emits green-yellow fluorescence; therefore, Ca^2+^ probes emitting other colours need to be chosen for simultaneous imaging. However, compared with GFP-based Ca^2+^ probes, such as the GCaMP series, only a few red or cyan Ca^2+^ probes have been developed that can be used in neurons *in vivo* [20–22]. Inverse-pericam fused with DsRed2 has been used for monitoring the intracellular Ca^2+^ concentration in pharyngeal muscles of *C*. *elegans* [50]. Furthermore, Hasen et al. succeeded in production of transgenic mouse with inverse-pericam and showed the change of intracellular calcium concentration [26]. This report suggests that IP2.0, which is a more sensitive indicator, may be functional in mammalian systems. We also propose that development of Ca^2+^ probes may be helpful for dual-colour imaging together with inverse-type Ca^2+^ probes in the same region to measure simultaneously neuronal excitatory and inhibitory activities.

We succeeded in the detection of neuronal inhibition in *C*. *elegans* with high sensitivity and we suggest that IP2.0 may enable the detection of neuronal inhibition in other species. Although conventional GECIs, such as GCaMPs, are useful for the detection of spike firing, they are barely capable of detecting neuronal inhibition [51, 52]. Therefore, the coexpression of IP2.0 with conventional one fluorophore GECIs, such as RCaMPs, may lead to sensitive and simultaneous detection of neuronal inhibition and excitation. Another quenching type GECI, Y-GECO1, may be able to detect neuronal inhibition *in vivo* and differences in its fluorescence characteristics, including the emission colour, may be useful for the analysis of neuronal activities. Y-GECO1 has a main excitation peak at 525 nm and an additional excitation peak around 413 nm in the presence of Ca^2+^; therefore, it might be difficult to use Y-GECO1 with other fluorescent proteins, including CFP, because of cross excitation, especially for monitoring fast changes of higher Ca^2+^ concentrations.

We predict that this kind of application will be helpful for understanding fast spiking neurons in the mammalian cortex, where action potentials are produced in the resting state and that inverse-type GECIs, such as IP2.0, will help unravel neuronal circuit activities in the brain.

## Materials and methods

### Construction of an inverse-pericam mutants library

The TorA protein export plasmid (pTorPE) (Invitrogen) was constructed by inserting a DNA fragment encoding TorA-6xHis-inverse-pericam into EcoRI and HindIII sites. Primers FW-CCTCGCCACAGAATTCATGGTCGACTCATCAATGAA and RW-CAAAACAGCCAAGCTTGTTACCATTCGCACGCTTAC were used for error-prone PCR to introduce random mutations into inverse-pericam. The resulting PCR products were digested with EcoRI and HindIII and ligated with similarly digested pTorPE.

### Screening of inverse-pericam mutant library

Competent One Shot Top10 *E*. *coli* (Invitrogen) were transformed with plasmids encoding the mutant library and cultured on nitrocellulose filters (ADVANTEC) overlaying LB-agar supplemented with 0.1 mM CaCl_2_, 0.0016% (wt/vol) L-arabinose and 50 μg/ml carbenicillin overnight at 37°C. Filters were transferred onto 1.5% agarose supplemented with 10 mM EGTA, 0.0016% L-arabinose and 50μg/ml carbenicillin (EGTA-agarose plate) and incubated for 4 h at 4°C. Before and after EGTA-agarose plate incubation, images were captured and colonies exhibiting a greater than ten times change in fluorescence intensity between the two images were picked. To compare images, we developed an image processing procedure using the MATLAB program and screened approximately 20,000 colonies (20 nitrocellulose membrane filters).

### Protein purification and characterisation of purified proteins

Recombinant fluorescent proteins with a polyhistidine tag at the N-terminus expressed in One Shot Top10 *E*. *coli* were subjected to Ni-NTA Sepharose purification and eluted as described [19]. The purified proteins in 30 mM MOPS (pH7.2) and 100 mM KCl were concentrated using Amicon Ultra-15 Centrifugal Filter Devices (Millipore) to make final concentration of 8 μg/μl and used for *in vitro* characterization. Purified proteins of IP2.0 and inverse pericam were characterized in 30 mM MOPS (pH7.2) and 100 mM KCl containing either 10 mM EGTA (Ca^2+^-free buffer) or 10 mM CaEGTA(Ca^2+^ buffer) (Molecular Probes by Life Technologies). Fluorescence emission (488 nm excitation) and excitation (515 nm emission) spectra were measured with 5 nm slits (JASCO FP-8200. Fluorescence Spectrometer).

For pH titrations, a solution containing 30 mM trisodium citrate and 30 mM borax was adjusted to pH 11.5 and HCl was then added dropwise to make solutions with pH values ranging from 11.5 to 4. One μl of concentrated protein in Ca^2+^-free buffer (30 mM MOPS (pH7.2), 100 mM KCl, 10 mM EGTA) or Ca^2+^-containing buffer (30 mM MOPS (pH7.2), 100 mM KCl, 10 mM CaCl_2_) was added into 100 μl of each of the buffers described above. The fluorescent intensities were normalized to the maximum value in Ca^2+^-free buffer (S2 File). Ca^2+^ titrations were performed by reciprocal dilution of a 1 μl of concentrated protein solution into a series of 100 μl of buffers mixed with Ca^2+^-free and Ca^2+^-saturated (39 μM) buffers (Molecular Probes by Life Technologies). The fluorescent intensities were normalized to the maximum value (S3 File). The Ca^2+^-titration fluorescence was fit to the Hill’s equation to extract the Hill coefficient and K_d_ for inverse-pericam and IP2.0.

k_off_ was determined from a single exponential fit to the fluorescence increase following rapid mixing of the protein samples in 30 mM MOPS (pH 7.2), 100 mM KCl, 10 mM EGTA•Ca^2+^, 10 mM KOH and buffer with 10 mM MOPS (pH 7.2), 100 mM KCl and 10 mM EGTA using a stopped-flow device coupled to a fluorometer (JASCO J1500 with SFC). The fluorescent intensities were normalized to the maximum value and were fit to the equation of 1-exp (-t/tau). These raw data can be accessed in figshare (https://doi.org/10.6084/m9.figshare.5976067.v1).

### HeLa cell culture and imaging

The culture and imaging of HeLa cells were performed as described before [8, 18]. Briefly, HeLa cells (40–60% confluent) grown on collagen-coated 35-mm glass bottom dishes (Mastumami) were transfected with 1 μg of plasmid DNA and 4 μL SuperFect Transfection Reagent (Qiagen) according to the manufacturer’s instructions. After incubation for 3 h the media was exchanged to DMEM containing 10% foetal bovine serum and the cells were incubated for an additional 24 h at 37°C in a CO_2_ incubator. Immediately prior to imaging, cells were washed twice with Hank’s balanced salt solution (HBSS) and then 1 mL of 20 mM HEPES buffered HBSS (HHBSS) was added.

Cell imaging was performed with an inverted Eclipse Ti-E microscope (Nikon) equipped with an electron multiplying (EM) iXon3 CCD camera (Andor). MetaMorph imaging software (Molecular Devices) was used for automated microscope and camera control. For determination of dynamic ranges in live cells, cells were imaged with a Plan Apo 60× 1.40 NA oil-immersion objective lens (Nikon). For excitation the samples were illuminated with light from a 100 W mercury arc lamp that was passed through 25% and 12.5% neutral density filters and a 497/16 nm bandpass filter. The emission filter was 535/22 nm. All imaging was performed at room temperature.

For imaging of histamine-induced Ca^2+^ dynamics, cells were imaged with a 100 ms exposure (2×2 binning) acquired every 5 s for a duration of 20 min. Approximately 30 s after the start of the experiment, histamine was added to a final concentration of 5 μM. Once the measurement had ended, cells were washed twice with HHBSS, and then incubated for 10 min in 1 mL HHBSS to allow histamine-induced oscillations to subside. Cells were then imaged as described above, with exposures every 10 s for a duration of 10 min. Approximately 1 min after imaging was started, 1 mL of 2 mM CaCl_2_, 10 μM ionomycin in Ca^2+^- and Mg^2+^-free HHBSS [HHBSS(-)] was added to the dish via a peristaltic pump. After measurements were completed, cells were washed 3 times with HHBSS(-) and 1 mL of HHBSS(-) was added. Approximately 2 min after imaging was started, 1 mL of 2 mM EGTA and 10 μM ionomycin in HHBSS(-) was added and cells were imaged with exposures every 10 s for a total of 8 min. These raw data can be accessed in figshare (https://doi.org/10.6084/m9.figshare.5976610.v1).

### Preparation and imaging of transgenic *C*. *elegans*

cDNA sequences encoding IP2.0 and GCaMP6f were optimized and three introns inserted for effective expression in *C*. *elegans* [53]. The modified cDNAs encoding IP2.0, GCaMP6f and mCherry (donated by Dr. Jorgensen, University of Utah) were used to construct a destination vector using the Gateway system (Invitrogen). Each destination vector was used to make an expression plasmid fragment by an LR reaction with an entry vector containing the *str-2* promotor or *gcy-7* promotor. We used the following transgenic worms that were constructed by microinjection of the DNA mixture [54]. N2; *qjEx11*[20 ng/μl *pstr-2*::GCaMP6f, 5 ng/μl *pstr-2*::mCherry, 5 ng/μl *plin-44*::*gfp*], N2; *qjEx12*[20 ng/μl *pstr-2*::GCaMP6f, 5 ng/μl *pstr-2*::mCherry, 5 ng/μl *plin-44*::*gfp*], N2; *qjEx15*[20 ng/μl *pstr-2*::IP2.0, 5 ng/μl *pstr-2*::mCherry, 5 ng/μl *plin-44*::*gfp*], N2; *qjEx17*[20 ng/μl *pstr-2*::IP2.0, 5 ng/μl *pstr-2*::mCherry, 5 ng/μl *plin-44*::*rfp*], N2; *qjEx21*[20 ng/μl *pstr-2*::IP2.0, 75 ng/μl *pstr-2*::RCaMP2.0, 5 ng/μl *plin-44*::*gfp*], N2; *qjEx22*[20 ng/μl *pgcy-7*::IP2.0, 10 ng/μl *pgcy-7*::mCherry, 5 ng/μl *plin-44*::*gfp*], N2; *qjEx23*[20 ng/μl *pgcy-7*::IP2.0, 75 ng/μl *pgcy-7*::mCherry, 5 ng/μl *plin-44*::*gfp*]. A young adult hermaphrodite expressing fluorescent proteins in an AWC^ON^ neuron or an ASEL neuron was put into a polydimethylsiloxane (PDMS) microfluidic chip for Ca^2+^ imaging. For imaging Ca^2+^ responses of an AWC^ON^ neuron, 0.001% IAA was added in imaging buffer (50 mM NaCl, 1 mM CaCl_2_, 1 mM MgCl_2_, 0.001% gelatine and 25 mM HEPES. pH 6.0) and perfused onto the top of the worm’s head. A buffer (25 mM NaCl, 1 mM CaCl_2_, 1 mM MgCl_2_, 0.001% gelatine and 25 mM HEPES. pH 6.0) was prepared and used for the analysis of ASEL neurons. Optical recordings were performed on a Zeiss Axioplan upright compound microscope fitted with an image splitting optics W-VIEW GEMINI (Hamamatsu Photonics) with bandpass filters, FF01-512/25 and FF01-650/60-25 (Semrock) and a dichroic mirror, FF580-FDi01-25x36 (Semrock). Fluorescence images were acquired using HSImage/HSR software (Hamamatsu Photonics) at 5 frames/second. The fluorescence intensity ratios of IP2.0 against mCherry were calculated using our MATLAB program. For imaging Ca^2+^ responses from two kinds of Ca^2+^ probe (IP2.0 and RCaMP2.0), an Olympus BX53-F microscope equipped with a 60× objective and an ORCA-D2 (Hamamatsu) was used with bandpass filters, FF01-650/60-25 (Semrock) and a dichroic mirror, 570 (Hamamatsu Photonics). The fluorescence intensity in a period before stimulation (time = 1–10 s for Fig 4A and 4B, time = 1–25 s for Fig 4C and time = 1–30 s for Fig 4D) was averaged and defined as *F*_0_. The change in fluorescence value of ROI relative to *F*_0_ was plotted for all stacks. All time series data relative to *F*_0_ can be accessed in figshare (https://doi.org/10.6084/m9.figshare.5976619.v1).

## Supporting information

S1 Fig Representative Ca^2+^ imaging in HeLa cells expressing IP2.0. Each dot was the ratio of fluorescent intensity of the first spike responding to histamine to the initial fluorescent intensity.
(TIFF)

S2 Fig *C*. *elegans* expressing GCaMP6f in an AWC^ON^ neuron. The region of interest (ROI) was defined by a square.
(TIFF)

S3 Fig Effects of transgenic genes of GCaMP6f or IP2.0 in AWC^ON^ on chemotaxis toward IAA. Chemotaxis toward IAA was analyzed on 9 cm chemotaxis assay plates as described previously (Bargmann CI et al, 1993), except that the assay plates contained 50 mM NaCl. The chemotaxis index was calculated as (A–B) / N, where A was the number of animals within 1.5 cm of the IAA spot, B was the number of animals within 1.5 cm of the control spot, and N was the number of all animals. A, 0.033%-0.33% IAA was spotted on assay plates, and 2 μl of 1 M sodium azide were placed on both the IAA spot and the control spot to anesthetize animals when they reached either spot. In the behavioral assays, the chemotaxis indexes of both the transgenic animals (*qjEx15* and *qjEx17*) and wild type animals were measured on the same assay plates. To distinguish these two, when we counted the number of animals on assay plates, P*lin44*::*gfp* or p*lin44*::*rfp* was used as injection markers for carrying transgenes. Prior to the behavioral assays, adult worms were washed twice with S-basal buffer (100 mM NaCl, 50 mM K_2_HPO_4_ [pH 6]) containing 0.02% gelatin, and once with water containing 0.02% gelatin. B, Chemotaxis indexes of warms expressing GCaMP6f (*qjEx12*) or IP2.0 (*qjEx15*) toward 0.33% IAA was analyzed. Error bars represent SEM (n = 4).
(TIFF)

S4 Fig Concentration dependency of IAA on IP2.0 Ca^2+^ responses in AWC^ON^. IP2.0 Ca^2+^ imaging was performed as described in Fig 4A (see Materials and methods). Bar graphs show fluorescence changes during the 60 seconds after stimulation of various concentration of IAA (10–70 sec. in Fig 4). The values are shown as relative to *F*_0_ (see Materials and methods) and error bars represent SEM (n = 5). These raw data can be accessed in figshare (https://doi.org/10.6084/m9.figshare.5976634.v1).
(TIFF)

S5 Fig *In vivo* imaging of Ca^2+^ responses in *C*.*elegans*. (A) GCaMP6f Ca^2+^ response to isoamylalcohol in AWC^ON^ (*qjEx11*) (n = 6). (B) IP2.0 Ca^2+^ response to isoamylalcohol in AWC^ON^ (*qjEx17*) (n = 7). (C) Dual-colour of RCaMP2.0 and IP2.0 Ca^2+^ responses to change of NaCl concentration (*qjEx23*) (n = 10). The values are shown as relative to *F*_*0*_ and error bars represent SEM. These raw data can be accessed in figshare (https://doi.org/10.6084/m9.figshare.5976643.v1).
(TIFF)

S1 Table Transgenic worms used in this work. (DOCX)

S1 File Data of excitation and emission spectra in Ca^2+^-free and Ca^2+^-saturated states of inverse-pericam and IP2.0. (PDF)

S2 File Data of fluorescent spectra under the various pH-condition at the 515 nm excitation peak in Ca^2+^-free and Ca^2+^-saturated states. (PDF)

S3 File Data of fluorescent spectra under the various Ca^2+^-concentration of inverse-pericam and IP2.0. (PDF)

S4 File Data of fluorescent intensity-change of IP2.0 after histamine stimulation in HeLa cells. (PDF)

S5 File Data of chemotaxis assay toward IAA using transgenic worms shown in S2 Fig. (PDF)

## References

[1] Scanziani M, Hausser M. Electrophysiology in the age of light. Nature. 2009; 461: 930–939 doi: 10.1038/nature08540 19829373 10.1038/nature08540

[2] Piet R, de Croft S, Liu X, Herbison AE. Electrical properties of kisspeptin neurons and their regulation of GnRH neurons. Front Neuroendocrinol. 2015; 36: 15–27 doi: 10.1016/j.yfrne.2014.05.006 24907402 10.1016/j.yfrne.2014.05.006

[3] Grynkiewicz G, Poenie M, Tsien RY. A new generation of Ca2+ indicators with greatly improved fluorescence properties. J Biol Chem. 1985; 260: 3440–3450 3838314

[4] Michalet X, Pinaud FF, Bentolila LA, Tsay JM, Doose S, Li JJ, Sundaresan G, et al Quantum dots for live cells, in vivo imaging, and diagnostics. Science. 2005; 307: 538–544 doi: 10.1126/science.1104274 15681376 10.1126/science.1104274 PMC1201471

[5] Tsien RY. Fluorescent probes of cell signaling. Annu Rev Neurosci. 1989; 12: 227–253 doi: 10.1146/annurev.ne.12.030189.001303 2648950 10.1146/annurev.ne.12.030189.001303

[6] Sohya K, Kameyama K, Yanagawa Y, Obata K, Tsumoto T. GABAergic neurons are less selective to stimulus orientation than excitatory neurons in layer II/III of visual cortex, as revealed by in vivo functional Ca2+ imaging in transgenic mice. J Neurosci. 2007; 27: 2145–2149 doi: 10.1523/JNEUROSCI.4641-06.2007 17314309 10.1523/JNEUROSCI.4641-06.2007 PMC6673543

[7] Baird GS, Zacharias DA, Tsien RY. Circular permutation and receptor insertion within green fluorescent proteins. Proc Natl Acad Sci U S A. 1999; 96: 11241–11246 10500161 10.1073/pnas.96.20.11241 PMC18018

[8] Nagai T, Sawano A, Park ES, Miyawaki A. Circularly permuted green fluorescent proteins engineered to sense Ca2+. Proc Natl Acad Sci U S A. 2001; 98: 3197–3202 doi: 10.1073/pnas.051636098 11248055 10.1073/pnas.051636098 PMC30630

[9] Grienberger C, Konnerth A. Imaging calcium in neurons. Neuron. 2012; 73: 862–885 doi: 10.1016/j.neuron.2012.02.011 22405199 10.1016/j.neuron.2012.02.011

[10] Broussard GJ, Liang R, Tian L. Monitoring activity in neural circuits with genetically encoded indicators. Front Mol Neurosci. 2014; 7: 97 doi: 10.3389/fnmol.2014.00097 25538558 10.3389/fnmol.2014.00097 PMC4256991

[11] Rose T, Goltstein PM, Portugues R, Griesbeck O. Putting a finishing touch on GECIs. Front Mol Neurosci. 2014; 7: 88 doi: 10.3389/fnmol.2014.00088 25477779 10.3389/fnmol.2014.00088 PMC4235368

[12] Whitaker M. Genetically encoded probes for measurement of intracellular calcium. Methods Cell Biol. 2010; 99: 153–182 doi: 10.1016/B978-0-12-374841-6.00006-2 21035686 10.1016/B978-0-12-374841-6.00006-2 PMC3292878

[13] Miyawaki A, Llopis J, Heim R, McCaffery JM, Adams JA, Ikura M, et al Fluorescent indicators for Ca2+ based on green fluorescent proteins and calmodulin. Nature. 1997; 388: 882–887 doi: 10.1038/42264 9278050 10.1038/42264

[14] Miyawaki A, Griesbeck O, Heim R, Tsien RY. Dynamic and quantitative Ca2+ measurements using improved cameleons. Proc Natl Acad Sci U S A. 1999; 96: 2135–2140 10051607 10.1073/pnas.96.5.2135 PMC26749

[15] Nagai T, Yamada S, Tominaga T, Ichikawa M, Miyawaki A. Expanded dynamic range of fluorescent indicators for Ca(2+) by circularly permuted yellow fluorescent proteins. Proc Natl Acad Sci U S A. 2004; 101: 10554–10559 doi: 10.1073/pnas.0400417101 15247428 10.1073/pnas.0400417101 PMC490022

[16] Akerboom J, Chen TW, Wardill TJ, Tian L, Marvin JS, Mutlu S, et al Optimization of a GCaMP calcium indicator for neural activity imaging. J Neurosci. 2012; 32: 13819–13840 doi: 10.1523/JNEUROSCI.2601-12.2012 23035093 10.1523/JNEUROSCI.2601-12.2012 PMC3482105

[17] Sun XR, Badura A, Pacheco DA, Lynch LA, Schneider ER, Taylor MP, et al Fast GCaMPs for improved tracking of neuronal activity. Nat Commun. 2013; 4: 2170 doi: 10.1038/ncomms3170 23863808 10.1038/ncomms3170 PMC3824390

[18] Chen TW, Wardill TJ, Sun Y, Pulver SR, Renninger SL, Baohan A, et al Ultrasensitive fluorescent proteins for imaging neuronal activity. Nature. 2013; 499: 295–300 doi: 10.1038/nature12354 23868258 10.1038/nature12354 PMC3777791

[19] Zhao Y, Araki S, Wu J, Teramoto T, Chang YF, Nakano M, et al An expanded palette of genetically encoded Ca(2)(+) indicators. Science. 2011; 333: 1888–1891 doi: 10.1126/science.1208592 21903779 10.1126/science.1208592 PMC3560286

[20] Akerboom J, Carreras Calderon N, Tian L, Wabnig S, Prigge M, Tolo J, et al Genetically encoded calcium indicators for multi-color neural activity imaging and combination with optogenetics. Front Mol Neurosci. 2013; 6: 2 doi: 10.3389/fnmol.2013.00002 23459413 10.3389/fnmol.2013.00002 PMC3586699

[21] Inoue M, Takeuchi A, Horigane S, Ohkura M, Gengyo-Ando K, Fujii H, et al Rational design of a high-affinity, fast, red calcium indicator R-CaMP2. Nat Methods. 2015; 12: 64–70 doi: 10.1038/nmeth.3185 25419959 10.1038/nmeth.3185

[22] Dana H, Mohar B, Sun Y, Narayan S, Gordus A, Hasseman JP, et al Sensitive red protein calcium indicators for imaging neural activity. Elife 5: 2016; 10.7554/eLife.12727 PMC4846379 27011354

[23] Laviv T, Kim BB, Chu J, Lam AJ, Lin MZ, Yasuda R. Simultaneous dual-color fluorescence lifetime imaging with novel red-shifted fluorescent proteins. Nat Methods. 2016; 13: 989–992 doi: 10.1038/nmeth.4046 27798609 10.1038/nmeth.4046 PMC5322478

[24] Boron WF, De Weer P. Intracellular pH transients in squid giant axons caused by CO2, NH3, and metabolic inhibitors. J Gen Physiol. 1976; 67: 91–112 1460 10.1085/jgp.67.1.91 PMC2214912

[25] Zhao Y, Abdelfattah AS, Zhao Y, Ruangkittisakul A, Ballanyi K, Campbell RE, et al Microfluidic cell sorter-aided directed evolution of a protein-based calcium ion indicator with an inverted fluorescent response. Integr Biol (Camb). 2014; 6: 714–725 24840546 10.1039/c4ib00039k

[26] Hasan MT, Friedrich RW, Euler T, Larkum ME, Giese G, Both M, et al Functional fluorescent Ca2+ indicator proteins in transgenic mice under TET control. PLoS Biol. 2004; 2: e163 doi: 10.1371/journal.pbio.0020163 15208716 10.1371/journal.pbio.0020163 PMC423138

[27] Helassa N, Zhang XH, Conte I, Scaringi J, Esposito E, Bradley J, et al Fast-Response Calmodulin-Based Fluorescent Indicators Reveal Rapid Intracellular Calcium Dynamics. Sci Rep. 2015; 5: 15978 doi: 10.1038/srep15978 26527405 10.1038/srep15978 PMC4630588

[28] Chalasani SH, Chronis N, Tsunozaki M, Gray JM, Ramot D, Goodman MB, et al Dissecting a circuit for olfactory behaviour in Caenorhabditis elegans. Nature. 2007; 450: 63–70 doi: 10.1038/nature06292 17972877 10.1038/nature06292

[29] Tian L, Hires SA, Mao T, Huber D, Chiappe ME, Chalasani SH, et al Imaging neural activity in worms, flies and mice with improved GCaMP calcium indicators. Nat Methods. 2009; 6: 875–881 doi: 10.1038/nmeth.1398 19898485 10.1038/nmeth.1398 PMC2858873

[30] Yoshida K, Hirotsu T, Tagawa T, Oda S, Wakabayashi T, Iino Y, et al Odour concentration-dependent olfactory preference change in C. elegans. Nat Commun. 2012; 3: 739 doi: 10.1038/ncomms1750 22415830 10.1038/ncomms1750

[31] Chronis N, Zimmer M, Bargmann CI. Microfluidics for in vivo imaging of neuronal and behavioral activity in Caenorhabditis elegans. Nat Methods. 2007; 4: 727–731 doi: 10.1038/nmeth1075 17704783 10.1038/nmeth1075

[32] Chalasani SH, Kato S, Albrecht DR, Nakagawa T, Abbott LF, Bargmann CI. Neuropeptide feedback modifies odor-evoked dynamics in Caenorhabditis elegans olfactory neurons. Nat Neurosci. 2010; 13: 615–621 doi: 10.1038/nn.2526 20364145 10.1038/nn.2526 PMC2937567

[33] Akerboom J, Chen TW, Wardill TJ, Tian L, Marvin JS, Mutlu S, et al Optimization of a GCaMP calcium indicator for neural activity imaging. J Neurosci. 2012; 32: 13819–13840 doi: 10.1523/JNEUROSCI.2601-12.2012 23035093 10.1523/JNEUROSCI.2601-12.2012 PMC3482105

[34] Suzuki H, Thiele TR, Faumont S, Ezcurra M, Lockery SR, Schafer WR. Functional asymmetry in Caenorhabditis elegans taste neurons and its computational role in chemotaxis. Nature. 2008; 454: 114–117 doi: 10.1038/nature06927 18596810 10.1038/nature06927 PMC2984562

[35] Nagai T, Horikawa K, Saito K, Matsuda T. Genetically encoded Ca(2+) indicators; expanded affinity range, color hue and compatibility with optogenetics. Front Mol Neurosci. 2014; 7: 90 doi: 10.3389/fnmol.2014.00090 25505381 10.3389/fnmol.2014.00090 PMC4243560

[36] Ohkura M, Sasaki T, Sadakari J, Gengyo-Ando K, Kagawa-Nagamura Y, Kobayashi C, et al Genetically encoded green fluorescent Ca2+ indicators with improved detectability for neuronal Ca2+ signals. PLoS One. 2012; 7: e51286 doi: 10.1371/journal.pone.0051286 23240011 10.1371/journal.pone.0051286 PMC3519846

[37] Helassa N, Podor B, Fine A, Torok K. Design and mechanistic insight into ultrafast calcium indicators for monitoring intracellular calcium dynamics. Sci Rep. 2016; 6: 38276 doi: 10.1038/srep38276 27922063 10.1038/srep38276 PMC5138832

[38] Nakai J, Ohkura M, Imoto K. A high signal-to-noise Ca(2+) probe composed of a single green fluorescent protein. Nat Biotechnol. 2001; 19: 137–141 doi: 10.1038/84397 11175727 10.1038/84397

[39] Tallini YN, Ohkura M, Choi BR, Ji G, Imoto K, Doran R, Lee J, et al Imaging cellular signals in the heart in vivo: Cardiac expression of the high-signal Ca2+ indicator GCaMP2. Proc Natl Acad Sci U S A. 2006; 103: 4753–4758 doi: 10.1073/pnas.0509378103 16537386 10.1073/pnas.0509378103 PMC1450242

[40] Muto A, Ohkura M, Kotani T, Higashijima S, Nakai J, Kawakami K. Genetic visualization with an improved GCaMP calcium indicator reveals spatiotemporal activation of the spinal motor neurons in zebrafish. Proc Natl Acad Sci U S A. 2011; 108: 5425–5430 doi: 10.1073/pnas.1000887108 21383146 10.1073/pnas.1000887108 PMC3069178

[41] Yamada Y, Mikoshiba K. Quantitative comparison of novel GCaMP-type genetically encoded Ca(2+) indicators in mammalian neurons. Front Cell Neurosci. 2012; 6: 41 doi: 10.3389/fncel.2012.00041 23060748 10.3389/fncel.2012.00041 PMC3465963

[42] van Giesen L, Neagu-Maier GL, Kwon JY, Sprecher SG. A microfluidics-based method for measuring neuronal activity in Drosophila chemosensory neurons. Nat Protoc. 2016; 11: 2389–2400 doi: 10.1038/nprot.2016.144 27809317 10.1038/nprot.2016.144

[43] Larsch J, Ventimiglia D, Bargmann CI, Albrecht DR. High-throughput imaging of neuronal activity in Caenorhabditis elegans. Proc Natl Acad Sci U S A. 2013; 110: E4266–4273 doi: 10.1073/pnas.1318325110 24145415 10.1073/pnas.1318325110 PMC3831453

[44] Kato S, Xu Y, Cho CE, Abbott LF, Bargmann CI. Temporal responses of C. elegans chemosensory neurons are preserved in behavioral dynamics. Neuron. 2014; 81: 616–628 doi: 10.1016/j.neuron.2013.11.020 24440227 10.1016/j.neuron.2013.11.020 PMC4112952

[45] Cormack BP, Valdivia RH, Falkow S. FACS-optimized mutants of the green fluorescent protein (GFP). Gene. 1996; 173: 33–38 8707053 10.1016/0378-1119(95)00685-0

[46] Arpino JA, Rizkallah PJ, Jones DD. Crystal structure of enhanced green fluorescent protein to 1.35 A resolution reveals alternative conformations for Glu222. PLoS One. 2012; 7: e47132 doi: 10.1371/journal.pone.0047132 23077555 10.1371/journal.pone.0047132 PMC3473056

[47] Kato S, Kaplan HS, Schrodel T, Skora S, Lindsay TH, Yemini E, et al Global brain dynamics embed the motor command sequence of Caenorhabditis elegans. Cell. 2015; 163: 656–669 doi: 10.1016/j.cell.2015.09.034 26478179 10.1016/j.cell.2015.09.034

[48] Nguyen JP, Shipley FB, Linder AN, Plummer GS, Liu M, Setru SU, et al Whole-brain calcium imaging with cellular resolution in freely behaving Caenorhabditis elegans. Proc Natl Acad Sci U S A. 2016; 113: E1074–1081 doi: 10.1073/pnas.1507110112 26712014 10.1073/pnas.1507110112 PMC4776509

[49] Branson K, Freeman J. Imaging the neural basis of locomotion. Cell. 2015; 163: 541–542 doi: 10.1016/j.cell.2015.10.014 26496599 10.1016/j.cell.2015.10.014

[50] Shimozono S, Fukano T, Kimura KD, Mori I, Kirino Y, Miyawaki A. Slow Ca2+ dynamics in pharyngeal muscles in Caenorhabditis elegans during fast pumping. EMBO Rep. 2004; 5: 521–526 doi: 10.1038/sj.embor.7400142 15088067 10.1038/sj.embor.7400142 PMC1299054

[51] Borghuis BG, Tian L, Xu Y, Nikonov SS, Vardi N, Zemelman BV, et al Imaging light responses of targeted neuron populations in the rodent retina. J Neurosci. 2011; 31: 2855–2867 doi: 10.1523/JNEUROSCI.6064-10.2011 21414907 10.1523/JNEUROSCI.6064-10.2011 PMC3521507

[52] Chen Q, Cichon J, Wang W, Qiu L, Lee SJ, Campbell NR, et al Imaging neural activity using Thy1-GCaMP transgenic mice. Neuron. 2012; 76: 297–308 doi: 10.1016/j.neuron.2012.07.011 23083733 10.1016/j.neuron.2012.07.011 PMC4059513

[53] Redemann S, Schloissnig S, Ernst S, Pozniakowsky A, Ayloo S, Hyman AA, et al Codon adaptation-based control of protein expression in C. elegans. Nat Methods. 2011; 8: 250–252 doi: 10.1038/nmeth.1565 21278743 10.1038/nmeth.1565

[54] Shinkai Y, Yamamoto Y, Fujiwara M, Tabata T, Murayama T, Hirotsu T, et al Behavioral choice between conflicting alternatives is regulated by a receptor guanylyl cyclase, GCY-28, and a receptor tyrosine kinase, SCD-2, in AIA interneurons of Caenorhabditis elegans. J Neurosci. 2011; 31: 3007–3015 doi: 10.1523/JNEUROSCI.4691-10.2011 21414922 10.1523/JNEUROSCI.4691-10.2011 PMC6623760

