# Supplementary figures and images for: An improved inverse-type Ca2+ indicator can detect putative neuronal inhibition in Caenorhabditis elegans by increasing signal intensity upon Ca2+ decrease
Source: PLoS One. 2018 Apr 25;13(4):e0194707. doi: 10.1371/journal.pone.0194707 (PMC5918796; doi:10.1371/journal.pone.0194707)

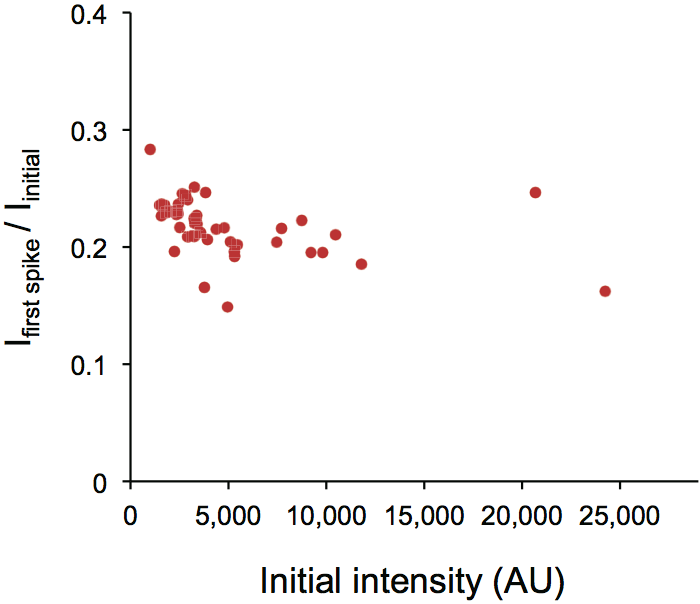

Supplement: S1 Fig — Each dot was the ratio of fluorescent intensity of the first spike responding to histamine to the initial fluorescent intensity. (TIFF) [file pone.0194707.s001.tiff]

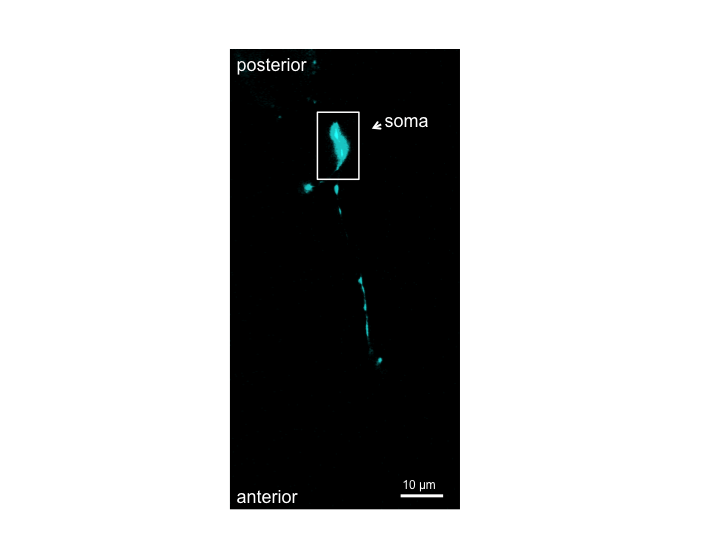

Supplement: S2 Fig — The region of interest (ROI) was defined by a square. (TIFF) [file pone.0194707.s002.tiff]

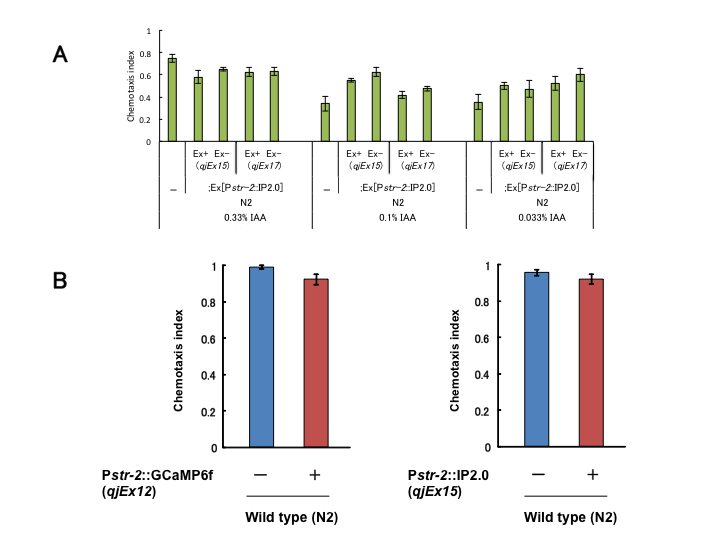

Supplement: S3 Fig — Chemotaxis toward IAA was analyzed on 9 cm chemotaxis assay plates as described previously (Bargmann CI et al, 1993), except that the assay plates contained 50 mM NaCl. The chemotaxis index was calculated as (A–B) / N, where A was the number of animals within 1.5 cm of the IAA spot, B was the number of animals within 1.5 cm of the control spot, and N was the number of all animals. A, 0.033%-0.33% IAA was spotted on assay plates, and 2 μl of 1 M sodium azide were placed on both the IAA spot and the control spot to anesthetize animals when they reached either spot. In the behavioral assays, the chemotaxis indexes of both the transgenic animals (qjEx15 and qjEx17) and wild type animals were measured on the same assay plates. To distinguish these two, when we counted the number of animals on assay plates, Plin44::gfp or plin44::rfp was used as injection markers for carrying transgenes. Prior to the behavioral assays, adult worms were washed twice with S-basal buffer (100 mM NaCl, 50 mM K2HPO4 [pH 6]) containing 0.02% gelatin, and once with water containing 0.02% gelatin. B, Chemotaxis indexes of warms expressing GCaMP6f (qjEx12) or IP2.0 (qjEx15) toward 0.33% IAA was analyzed. Error bars represent SEM (n = 4). (TIFF) [file pone.0194707.s003.tiff]

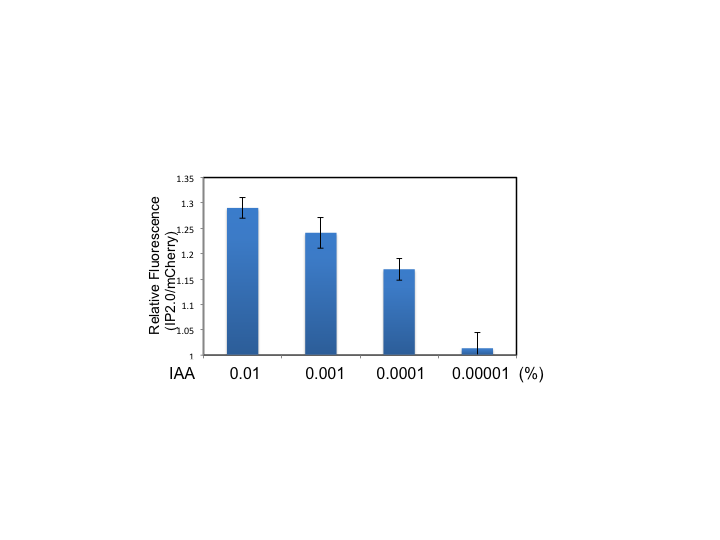

Supplement: S4 Fig — IP2.0 Ca2+ imaging was performed as described in Fig 4A (see Materials and methods). Bar graphs show fluorescence changes during the 60 seconds after stimulation of various concentration of IAA (10–70 sec. in Fig 4). The values are shown as relative to F0 (see Materials and methods) and error bars represent SEM (n = 5). These raw data can be accessed in figshare (https://doi.org/10.6084/m9.figshare.5976634.v1). (TIFF) [file pone.0194707.s004.tiff]

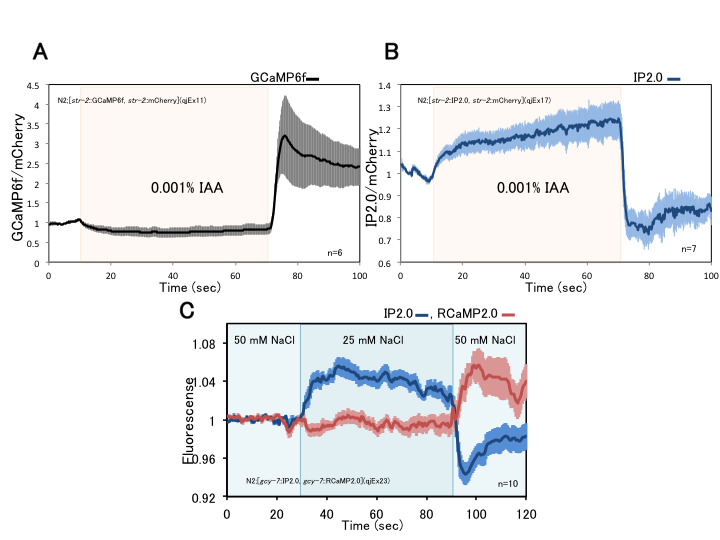

Supplement: S5 Fig — (A) GCaMP6f Ca2+ response to isoamylalcohol in AWCON (qjEx11) (n = 6). (B) IP2.0 Ca2+ response to isoamylalcohol in AWCON (qjEx17) (n = 7). (C) Dual-colour of RCaMP2.0 and IP2.0 Ca2+ responses to change of NaCl concentration (qjEx23) (n = 10). The values are shown as relative to F0 and error bars represent SEM. These raw data can be accessed in figshare (https://doi.org/10.6084/m9.figshare.5976643.v1). (TIFF) [file pone.0194707.s005.tiff]
