# Supplementary material for: An improved inverse-type Ca2+ indicator can detect putative neuronal inhibition in Caenorhabditis elegans by increasing signal intensity upon Ca2+ decrease
Source: PLoS One. 2018 Apr 25;13(4):e0194707. doi: 10.1371/journal.pone.0194707 (PMC5918796; doi:10.1371/journal.pone.0194707)
Supplement: S1 Table — (DOCX) [file pone.0194707.s006.docx]

| genotype |
| --- |
| N2; *qjEx11*[*pstr-2*::GCaMP6f, *pstr-2*::mCherry, *plin-44*::*gfp*]  N2; *qjEx12*[*pstr-2*::GCaMP6f, *pstr-2*::mCherry, *plin-44*::*gfp*]  N2; *qjEx15*[*pstr-2*::IP2.0, *pstr-2*::mCherry, *plin-44*::*gfp*]  N2; *qjEx17*[*pstr-2*::IP2.0, *pstr-2*::mCherry, *plin-44*::*rfp*]  N2; *qjEx21*[*pstr-2*::IP2.0, *pstr-2*::RCaMP2.0, *plin-44*::*gfp*]  N2; *qjEx22*[*pgcy-7*::IP2.0, *pgcy-7*::mCherry, *plin-44*::*gfp*]  N2; *qjEx23*[*pgcy-7*::IP2.0, *pgcy-7*::mCherry, *plin-44*::*gfp*] |
