# Supplementary material for: An improved inverse-type Ca2+ indicator can detect putative neuronal inhibition in Caenorhabditis elegans by increasing signal intensity upon Ca2+ decrease
Source: PLoS One. 2018 Apr 25;13(4):e0194707. doi: 10.1371/journal.pone.0194707 (PMC5918796; doi:10.1371/journal.pone.0194707)
Supplement: S1 File — (PDF) [file pone.0194707.s007.pdf]

	IP2.0 (Ex, +Ca)	IP2.0 (Ex, −Ca)	IP2.0 (Em,+Ca)	IP2.0 (Em, −Ca)
420	6.20271	13.6771		
420.5	6.324	13.7655		
421	6.27036	14.0845		
421.5	6.27412	14.282		
422	6.32649	14.4524		
422.5	6.42182	14.797		
423	6.4405	15.0829		
423.5	6.45911	15.4908		
424	6.43895	15.8738		
424.5	6.49426	16.2105		
425	6.50341	16.6062		
425.5	6.44006	17.0863		
426	6.48829	17.531		
426.5	6.57887	18.0402		
427	6.61219	18.4005		
427.5	6.71891	18.9131		
428	6.87243	19.4662		
428.5	7.00429	19.9719		
429	7.08617	20.3925		
429.5	7.10669	20.7798		
430	7.14617	21.2516		
430.5	7.21028	21.9287		
431	7.25018	22.5294		
431.5	7.26559	23.0429		
432	7.30686	23.6651		
432.5	7.38125	24.3562		
433	7.51085	25.2233		
433.5	7.77563	26.0558		
434	8.00658	26.7398		
434.5	8.27411	27.5205		
435	8.57119	28.3512		
435.5	8.90238	29.3745		
436	9.28804	30.5108		
436.5	9.62559	31.701		
437	10.0483	32.8011		
437.5	10.4916	33.9157		
438	10.7414	35.2747		
438.5	11.0939	36.7103		
439	11.3595	37.6511		
439.5	11.6308	38.8018		

440	11.8729	39.8476
440.5	11.877	41.1289
441	11.9259	42.2224
441.5	12.0183	43.2513
442	12.0874	44.0697
442.5	12.2379	45.0479
443	12.185	45.979
443.5	12.2057	46.8978
444	12.2446	47.7578
444.5	12.2654	48.5684
445	12.2718	49.5422
445.5	12.187	50.8475
446	12.1981	52.0989
446.5	12.2943	53.1694
447	12.2924	54.392
447.5	12.284	55.6758
448	12.4573	57.1136
448.5	12.7195	59.0043
449	12.9723	60.4512
449.5	13.046	61.7112
450	13.2171	63.3904
450.5	13.5841	65.0859
451	13.8505	66.8269
451.5	14.044	68.6614
452	14.2018	69.9267
452.5	14.4981	72.0006
453	14.9308	74.5382
453.5	15.1812	76.4132
454	15.364	78.3605
454.5	15.6382	80.6989
455	15.8865	83.074
455.5	16.1014	85.52
456	16.1597	87.5454
456.5	16.3055	89.8586
457	16.4151	92.6408
457.5	16.5351	95.3596
458	16.7221	98.0114
458.5	16.8386	100.844
459	17.029	104.021
459.5	17.3051	107.317
460	17.6114	110.713

460.5	17.9001	114.378
461	18.1743	118.189
461.5	18.5197	121.762
462	18.8844	125.37
462.5	19.0912	129.188
463	19.2907	133.333
463.5	19.4512	137.302
464	19.6275	141.268
464.5	19.7345	145.775
465	19.8991	150.908
465.5	20.1135	155.701
466	20.3486	160.795
466.5	20.5799	166.105
467	20.8378	171.674
467.5	21.1686	176.592
468	21.5008	181.414
468.5	21.6796	186.575
469	21.8841	191.374
469.5	22.0315	196.105
470	22.0964	200.023
470.5	22.2184	204.125
471	22.2749	208.629
471.5	22.4764	213.318
472	22.7951	217.536
472.5	23.107	221.501
473	23.4416	225.114
473.5	23.7812	228.103
474	24.1482	231.127
474.5	24.4191	233.803
475	24.5603	235.695
475.5	24.7591	237.685
476	24.8921	239.588
476.5	25.0855	241.713
477	25.4653	244.002
477.5	25.6723	245.216
478	26.0504	246.589
478.5	26.4267	248.226
479	26.8119	250.048
479.5	27.2119	251.805
480	27.4705	253.807
480.5	27.5679	255.877

481	27.9577	258.281
481.5	28.367	260.583
482	28.6495	262.914
482.5	28.9191	264.952
483	29.1452	267.208
483.5	29.2834	269.763
484	29.4386	272.975
484.5	29.4963	276.393
485	29.4377	279.938
485.5	29.4738	283.936
486	29.5541	288.444
486.5	29.7506	293.451
487	30.0017	298.586
487.5	30.2325	304.053
488	30.4085	309.992
488.5	30.5245	316.272
489	30.5273	323.026
489.5	30.6532	329.885
490	30.81	337.497
490.5	30.9327	345.402
491	30.8436	353.916
491.5	30.8822	362.403
492	30.8451	371.408
492.5	30.9049	380.724
493	30.7365	390.465
493.5	30.339	400.303
494	30.0676	411.012
494.5	29.8238	421.623
495	29.5399	432.841
495.5	29.4006	444.707
496	29.3238	456.795
496.5	29.1049	469.005
497	28.7889	480.955
497.5	28.4124	493.205
498	28.0725	505.96
498.5	27.7296	518.102
499	27.076	530.108
499.5	26.3687	541.993
500	25.884	553.54
500.5	25.4634	564.192
501	24.9764	573.874

501.5	24.465	583.301		
502	23.9711	591.951		
502.5	23.4591	599.001		
503	22.9917	605.15		
503.5	22.4024	609.165		
504	21.8682	612.587		
504.5	21.2206	613.769		
505	20.5844	612.627		
505.5	19.9871	610.117		
506	19.4493	605.79		
506.5	18.8032	599.177		
507	18.182	590.854		
507.5	17.6027	580.224		
508	16.9837	567.253		
508.5	16.2712	552.869		
509	15.5213	537.21		
509.5	14.8686	519.682		
510	14.2623	500.982	24.518	493.665
510.5	13.5241	481.015	24.9354	513.247
511	12.7492	459.993	25.136	531.247
511.5	12.1011	438.149	25.3962	548.097
512	11.5688	416.068	25.4634	563.52
512.5			25.5234	577.445
513			25.5918	589.313
513.5			25.657	599.36
514			25.7094	607.498
514.5			25.713	613.292
515			25.6382	616.99
515.5			25.606	618.282
516			25.4149	617.955
516.5			25.1322	615.637
517			24.7432	610.835
517.5			24.236	604.592
518			23.8508	596.684
518.5			23.2997	587.322
519			22.7331	576.557
519.5			22.23	564.629
520			21.7192	552.922
520.5			21.1914	539.914
521			20.6703	526.123
521.5			19.9964	511.711

522	19.549	497.085
522.5	18.9374	482.101
523	18.2078	466.501
523.5	17.5934	450.85
524	17.1547	435.684
524.5	16.6802	421.478
525	16.2303	407.561
525.5	15.7706	393.131
526	15.3088	379.099
526.5	14.97	365.669
527	14.5353	352.806
527.5	14.001	340.087
528	13.537	327.36
528.5	13.1523	315.957
529	12.7853	305.625
529.5	12.3813	295.51
530	11.974	285.922
530.5	11.6369	276.184
531	11.3483	266.594
531.5	11.0394	258.229
532	10.6804	249.744
532.5	10.3148	241.471
533	10.1046	234.051
533.5	9.84373	226.608
534	9.6292	220.286
534.5	9.35576	214.506
535	9.12531	208.17
535.5	8.94266	202.667
536	8.7671	197.39
536.5	8.4878	192.216
537	8.31506	187.376
537.5	8.15477	182.66
538	8.00549	178.118
538.5	7.8282	173.922
539	7.65565	170.068
539.5	7.46538	166.379
540	7.26717	162.872
540.5	7.05857	159.806
541	6.9232	156.759
541.5	6.79644	153.488
542	6.65698	150.262

542.5	6.55788	147.222
543	6.47285	144.262
543.5	6.42815	141.671
544	6.36628	139.361
544.5	6.2695	137.356
545	6.18039	135.435
545.5	6.13844	133.711
546	6.06615	132.08
546.5	6.09557	130.508
547	5.99662	128.583
547.5	5.90794	126.529
548	5.8855	124.73
548.5	5.83578	122.928
549	5.75331	120.972
549.5	5.66489	119.169
550	5.52029	117.634
550.5	5.45331	115.874
551	5.32071	113.864
551.5	5.19519	112.081
552	5.10486	110.291
552.5	5.00909	108.489
553	4.89079	106.642
553.5	4.80027	104.759
554	4.70513	102.988
554.5	4.57734	100.999
555	4.45516	98.8474
555.5	4.2734	96.7189
556	4.15303	94.6679
556.5	4.03218	92.6228
557	3.90741	90.6055
557.5	3.76779	88.6571
558	3.6913	86.9101
558.5	3.56794	85.1249
559	3.5694	83.1893
559.5	3.48187	81.1656
560	3.42221	79.0958
560.5	3.28982	76.9453
561	3.21715	74.8875
561.5	3.14139	72.9447
562	3.05816	71.1175
562.5	2.92648	69.3534

563	2.84744	67.4884
563.5	2.77671	65.6357
564	2.71266	63.8441
564.5	2.61907	61.8684
565	2.54381	59.8831
565.5	2.46073	58.0815
566	2.35999	56.4534
566.5	2.27865	54.8739
567	2.2287	53.0945
567.5	2.17828	51.6595
568	2.15225	50.2716
568.5	2.08553	48.9177
569	2.08475	47.5481
569.5	2.02385	46.0533
570	1.95726	44.7165
570.5	1.90229	43.5667
571	1.85481	42.172
571.5	1.82442	41.1511
572	1.75316	39.8709
572.5	1.70016	38.6408
573	1.69552	37.5018
573.5	1.6727	36.3059
574	1.64733	35.2214
574.5	1.604	34.1817
575	1.53731	33.1388
575.5	1.50969	32.0484
576	1.46441	31.0325
576.5	1.435	30.127
577	1.43897	29.2221
577.5	1.42901	28.3349
578	1.41746	27.2187
578.5	1.39647	26.2816
579	1.37401	25.606
579.5	1.33953	24.8163
580	1.26718	23.8984
580.5	1.20146	23.2002
581	1.15932	22.5206
581.5	1.13097	21.8986
582	1.12301	21.2723
582.5	1.09481	20.6083
583	1.09686	20.0196

583.5	1.09662	19.4604
584	1.08351	18.8895
584.5	1.05707	18.3407
585	1.00719	17.8325
585.5	0.999851	17.3484
586	0.962306	16.8772
586.5	0.922275	16.6083
587	0.884282	16.2787
587.5	0.92166	15.8625
588	0.937506	15.5346
588.5	0.963959	15.0835
589	0.925513	14.7068
589.5	0.924995	14.411
590	0.896572	13.9769
590.5	0.877578	13.6627
591	0.833509	13.2968
591.5	0.826406	12.9233
592	0.840261	12.6226
592.5	0.837431	12.2803
593	0.836294	11.9402
593.5	0.835659	11.6128
594	0.830612	11.3235
594.5	0.846035	11.11
595	0.85037	10.878
595.5	0.838095	10.6817
596	0.831692	10.4554
596.5	0.829042	10.2462
597	0.841327	9.96575
597.5	0.855234	9.71686
598	0.836511	9.49301
598.5	0.817035	9.29058
599	0.837136	9.00175
599.5	0.876148	8.74737
600	0.879332	8.61969

	Inverse (Ex, +C	Inverse (Ex, −C	Inverse (Em,+C	Inverse (Em, −Ca)
420	3.63154	3.81099		
420.5	3.65357	3.81954		
421	3.68793	3.86496		
421.5	3.65935	3.87468		
422	3.58974	3.8429		
422.5	3.61321	3.87929		
423	3.67233	3.89877		
423.5	3.66052	3.97796		
424	3.73019	4.04095		
424.5	3.79946	4.01859		
425	3.87946	4.03163		
425.5	4.0245	4.08072		
426	4.09926	4.12061		
426.5	4.17905	4.15967		
427	4.29477	4.21566		
427.5	4.38035	4.28348		
428	4.41434	4.43009		
428.5	4.45676	4.51056		
429	4.47784	4.66328		
429.5	4.51559	4.81903		
430	4.50008	4.94451		
430.5	4.4549	5.0561		
431	4.48217	5.06805		
431.5	4.56424	5.14877		
432	4.64005	5.32797		
432.5	4.77092	5.47697		
433	4.89174	5.65279		
433.5	5.0348	5.90244		
434	5.25728	6.22698		
434.5	5.49324	6.63009		
435	5.73453	7.02447		
435.5	6.00971	7.37172		
436	6.2836	7.72044		
436.5	6.62281	8.13972		
437	6.97639	8.61085		
437.5	7.26772	8.97114		
438	7.57717	9.36959		
438.5	7.75259	9.69237		
439	8.00781	10.0872		
439.5	8.14114	10.4176		

440	8.33496	10.758
440.5	8.47615	10.8872
441	8.49142	11.0813
441.5	8.46493	11.2483
442	8.48501	11.3754
442.5	8.44786	11.4528
443	8.38632	11.5703
443.5	8.22443	11.7077
444	8.11922	11.8485
444.5	8.00632	11.9192
445	7.92319	12.0615
445.5	7.85917	12.295
446	7.81062	12.5165
446.5	7.82671	12.6159
447	7.83213	12.6941
447.5	7.88425	12.856
448	7.94403	13.1697
448.5	8.03999	13.5238
449	8.16298	13.8805
449.5	8.27701	14.1873
450	8.36137	14.5385
450.5	8.45648	14.8904
451	8.61459	15.3211
451.5	8.75902	15.6469
452	8.8217	15.8798
452.5	8.84618	16.1852
453	8.98492	16.5101
453.5	9.11769	16.9092
454	9.28724	17.2337
454.5	9.43255	17.7165
455	9.64429	18.2353
455.5	9.80982	18.7537
456	10.0618	19.2747
456.5	10.2031	19.9402
457	10.3956	20.6569
457.5	10.5246	21.3338
458	10.6316	21.9547
458.5	10.7558	22.5316
459	10.8692	23.2202
459.5	10.9673	23.9856
460	11.0549	24.6605

460.5	11.1219	25.4034
461	11.1838	26.2057
461.5	11.3052	27.0252
462	11.4596	27.8811
462.5	11.6545	28.7135
463	11.7626	29.6131
463.5	11.8622	30.5277
464	11.9493	31.4749
464.5	12.191	32.4272
465	12.2848	33.587
465.5	12.3823	34.8182
466	12.3889	36.0547
466.5	12.5787	37.1916
467	12.8021	38.3354
467.5	12.9675	39.6179
468	13.0765	40.7213
468.5	13.2545	41.8567
469	13.4433	42.9665
469.5	13.6225	43.9753
470	13.6766	45.091
470.5	13.6797	46.0228
471	13.7107	47.0829
471.5	13.8182	48.115
472	13.892	49.1032
472.5	13.9853	50.1021
473	14.0998	50.948
473.5	14.2499	51.745
474	14.4721	52.5462
474.5	14.6663	53.2344
475	14.8056	53.8423
475.5	14.9013	54.218
476	14.9529	54.5046
476.5	15.1645	55.0146
477	15.3608	55.6261
477.5	15.5717	56.0799
478	15.8498	56.6613
478.5	16.0358	57.2895
479	16.2063	57.9676
479.5	16.5821	58.7442
480	16.6707	59.2226
480.5	16.8796	59.7013

481	17.0844	60.2573
481.5	17.2253	60.7964
482	17.5106	61.3377
482.5	17.7914	61.7931
483	17.9557	62.3189
483.5	18.1799	62.8559
484	18.3504	63.4277
484.5	18.4782	64.3185
485	18.6531	65.1639
485.5	18.8406	66.1912
486	19.0078	67.3465
486.5	19.2159	68.5103
487	19.3605	69.8384
487.5	19.4232	71.0773
488	19.4534	72.3768
488.5	19.3921	73.728
489	19.2711	75.1338
489.5	19.1508	76.6179
490	18.9591	78.0282
490.5	18.8539	79.9364
491	18.8856	81.8777
491.5	18.8063	83.8618
492	18.748	85.7546
492.5	18.8778	87.7612
493	18.7767	90.1198
493.5	18.7098	92.5878
494	18.5501	94.9641
494.5	18.3613	97.2036
495	18.2202	99.627
495.5	18.0635	102.23
496	17.8628	105.14
496.5	17.6419	107.948
497	17.4434	110.665
497.5	17.2113	113.502
498	16.8858	116.31
498.5	16.6094	119.109
499	16.2047	121.689
499.5	15.7954	124.071
500	15.3517	126.473
500.5	14.9099	128.948
501	14.4937	131.561

501.5	14.1719	134.197		
502	13.5656	136.385		
502.5	13.0214	138.148		
503	12.6338	139.83		
503.5	12.1257	140.976		
504	11.6333	141.155		
504.5	11.1682	140.827		
505	10.7352	140.392		
505.5	10.4479	139.771		
506	10.084	138.556		
506.5	9.7388	136.713		
507	9.42178	134.606		
507.5	9.09471	132.187		
508	8.68518	128.84		
508.5	8.17515	125.113		
509	7.7397	121.264		
509.5	7.29206	117.077		
510	6.8764	112.72	14.4092	126.72
510.5	6.5155	108.109	14.519	131.281
511	6.11293	103.422	14.6495	135.457
511.5	5.71426	98.3313	14.7907	139.065
512	5.48808	93.3545	14.8775	142.609
512.5			14.9129	145.705
513			14.9158	148.01
513.5			14.8776	149.861
514			14.8443	151.145
514.5			14.7526	152.06
515			14.6522	152.443
515.5			14.5609	152.061
516			14.4747	151.436
516.5			14.3353	150.346
517			14.0979	148.816
517.5			13.7779	147.111
518			13.3667	144.699
518.5			13.085	141.894
519			12.8493	138.724
519.5			12.6026	135.533
520			12.2749	132.569
520.5			12.0182	129.166
521			11.7948	125.354
521.5			11.722	121.757

522	11.3532	118.503
522.5	11.0758	115.058
523	10.7113	111.618
523.5	10.499	107.851
524	10.2307	104.089
524.5	9.92067	100.606
525	9.53343	97.3625
525.5	9.21877	93.8562
526	8.91316	90.6181
526.5	8.60418	87.2665
527	8.31647	84.0921
527.5	8.0427	81.1214
528	7.8285	78.3982
528.5	7.61872	75.6521
529	7.40393	72.9632
529.5	7.23321	70.4289
530	7.02939	68.0663
530.5	6.79469	65.8543
531	6.52048	63.6321
531.5	6.25938	61.448
532	6.13395	59.2696
532.5	6.00667	57.346
533	5.82575	55.7334
533.5	5.75864	54.1861
534	5.61068	52.5865
534.5	5.5356	51.2627
535	5.44092	49.9303
535.5	5.29725	48.8307
536	5.17089	47.6064
536.5	5.08498	46.3158
537	4.94422	44.9677
537.5	4.89192	43.9233
538	4.83127	42.7898
538.5	4.74334	41.7154
539	4.62492	40.6567
539.5	4.57472	39.6712
540	4.50549	38.8977
540.5	4.45923	38.2164
541	4.36614	37.3943
541.5	4.26402	36.7041
542	4.17272	36.1612

542.5	4.0795	35.6962
543	3.98844	35.1727
543.5	3.88999	34.5715
544	3.81126	34.1355
544.5	3.73105	33.7393
545	3.67609	33.114
545.5	3.71338	32.569
546	3.70635	31.886
546.5	3.71663	31.4736
547	3.65457	30.9297
547.5	3.63527	30.581
548	3.63787	30.0938
548.5	3.59107	29.8335
549	3.55098	29.3857
549.5	3.49276	28.8414
550	3.44373	28.4046
550.5	3.42274	28.0177
551	3.38779	27.4363
551.5	3.30904	26.8976
552	3.2015	26.3568
552.5	3.07471	25.9587
553	2.95054	25.6111
553.5	2.81214	25.1697
554	2.71929	24.7281
554.5	2.65642	24.4286
555	2.63943	24.0466
555.5	2.62432	23.6401
556	2.56107	23.1848
556.5	2.54809	22.6307
557	2.48863	22.1431
557.5	2.38166	21.599
558	2.33307	20.9937
558.5	2.31003	20.4014
559	2.26287	19.8418
559.5	2.24786	19.3307
560	2.19913	18.8336
560.5	2.1603	18.3397
561	2.06642	17.876
561.5	1.94889	17.4072
562	1.86312	16.9461
562.5	1.80426	16.4767

563	1.76497	16.0199
563.5	1.74364	15.5698
564	1.73289	15.0394
564.5	1.70702	14.5871
565	1.63789	14.2306
565.5	1.58754	13.9209
566	1.57916	13.593
566.5	1.51012	13.2208
567	1.41825	12.8387
567.5	1.36821	12.4924
568	1.34829	12.0697
568.5	1.33327	11.6903
569	1.29189	11.2386
569.5	1.22651	10.8428
570	1.18476	10.4616
570.5	1.21071	10.1432
571	1.18653	9.88431
571.5	1.15016	9.57185
572	1.14604	9.28188
572.5	1.13812	9.00489
573	1.09653	8.74008
573.5	1.03394	8.47755
574	0.977071	8.22165
574.5	0.957325	7.95978
575	0.965414	7.72132
575.5	0.925204	7.4826
576	0.873562	7.27275
576.5	0.854397	7.06306
577	0.845674	6.91418
577.5	0.841219	6.7635
578	0.836463	6.59408
578.5	0.77937	6.4221
579	0.777745	6.27885
579.5	0.803583	6.09047
580	0.7905	5.91866
580.5	0.801408	5.78637
581	0.778069	5.60629
581.5	0.743094	5.4178
582	0.739607	5.24569
582.5	0.713596	5.04215
583	0.686066	4.82908

583.5	0.680278	4.63347
584	0.662598	4.44982
584.5	0.668969	4.36791
585	0.712673	4.25255
585.5	0.694065	4.18352
586	0.716968	4.05987
586.5	0.73097	3.95051
587	0.745719	3.90046
587.5	0.718377	3.8506
588	0.728047	3.67357
588.5	0.654379	3.56664
589	0.651177	3.45075
589.5	0.631865	3.35617
590	0.59473	3.25684
590.5	0.577976	3.1412
591	0.576844	3.01292
591.5	0.55558	2.92705
592	0.565738	2.8517
592.5	0.538458	2.80854
593	0.519533	2.76324
593.5	0.558677	2.7178
594	0.557678	2.67526
594.5	0.559209	2.60224
595	0.553186	2.53493
595.5	0.55461	2.43885
596	0.562691	2.38913
596.5	0.589289	2.30938
597	0.579779	2.2147
597.5	0.612697	2.17797
598	0.607492	2.2183
598.5	0.625261	2.18059
599	0.673751	2.14596
599.5	0.694078	2.13844
600	0.686234	2.16699
