# Supplementary material for: An improved inverse-type Ca2+ indicator can detect putative neuronal inhibition in Caenorhabditis elegans by increasing signal intensity upon Ca2+ decrease
Source: PLoS One. 2018 Apr 25;13(4):e0194707. doi: 10.1371/journal.pone.0194707 (PMC5918796; doi:10.1371/journal.pone.0194707)
Supplement: S2 File — (PDF) [file pone.0194707.s008.pdf]

EGTA	Ex. 1	Ex. 2	Ex. 3			
pH	ratio1	raio2	ratio3	avg	sd	se
3	1.814581106	2.301114659	2.909169783	2.341621849	0.447780986	0.258526473
3.5	1.552867515	1.942857391	2.623178022	2.03963431	0.44227855	0.25534964
4	1.567164909	1.916648539	2.49496787	1.992927106	0.382594998	0.220891325
4.5	2.000670845	2.044059936	2.748040884	2.264257222	0.342545011	0.197768454
5	3.567430452	3.437806183	3.681615655	3.562284097	0.0996013	0.057504837
5.5	9.168710649	7.973077626	7.531233907	8.224340727	0.691704359	0.399355698
6	28.83381201	23.05778652	20.74894208	24.21351353	3.400299904	1.963164065
6.5	47.22891903	40.06730594	35.99752592	41.09791696	4.642748262	2.680491959
7	62.79183525	54.71837665	48.60807604	55.37276265	5.808954131	3.353801231
7.5	69.41783191	63.57213091	62.70850592	65.23282291	2.980177541	1.720606306
8	76.09658777	66.60024538	66.56180731	69.75288016	4.485706122	2.589823637
8.5	84.72743409	75.60148969	73.22448111	77.85113496	4.958168994	2.862600203
9	94.00466797	87.4502928	86.54423124	89.333064	3.323968469	1.91909409
9.5	97.94064415	99.21512665	99.44920852	98.86832644	0.662894864	0.382722528
10	100	100	99.5410071	99.84700237	0.216371329	0.124922045
10.5	95.85683039	88.93476502	100	94.9305318	4.564601342	2.635373814
11	90.08336653	84.42174325	77.62465576	84.04325518	5.093283711	2.940608721
11.5	83.97203413	74.45942066	70.32442962	76.25196147	5.713969965	3.298962098

Ca2+	Ex. 1	Ex. 2	Ex. 3			
pH	ratio1	raio2	ratio3	avg	sd	se
3	1.115567109	1.410487892	1.757518332	1.427857778	0.262363143	0.151475431
3.5	1.117359524	1.37922348	1.754369292	1.416984099	0.261425272	0.150933951
4	1.102869961	1.347832897	1.70477192	1.385158259	0.247138799	0.142685652
4.5	1.100784051	1.282188944	1.676301228	1.353091408	0.240243453	0.138704622
5	1.086186174	1.280131044	1.654454766	1.340257328	0.235858257	0.136172828
5.5	1.059789103	1.284999521	1.661669441	1.335486022	0.248296396	0.143353991
6	1.067084547	1.218220026	1.638720815	1.308008463	0.241851853	0.139633232
6.5	1.030967205	1.285486804	1.653886815	1.323446941	0.255718516	0.147639154
7	1.07533385	1.287988479	1.634925098	1.332749143	0.23063424	0.13315674
7.5	1.074603607	1.397087615	1.700307031	1.390666085	0.255482708	0.14750301
8	1.151562162	1.420807845	1.694098925	1.422156311	0.221491759	0.127878326
8.5	1.881896257	2.526822307	3.062553421	2.490423995	0.482687934	0.278680009
9	4.069481422	4.594902674	5.488494759	4.717626285	0.585773293	0.338196368
9.5	9.210463792	10.71473943	13.4940843	11.13976251	1.774417207	1.024460252
10	13.58207026	16.84562707	21.15423546	17.1939776	3.101121574	1.790433375
10.5	16.41041767	23.06352949	29.11320797	22.86238505	5.187842498	2.995202263
11	19.09952971	25.06660982	32.6708354	25.61232497	5.553883823	3.20653632
11.5	19.0652537	23.95895513	31.37978542	24.80133141	5.062550162	2.922864699
