# Supplementary material for: An improved inverse-type Ca2+ indicator can detect putative neuronal inhibition in Caenorhabditis elegans by increasing signal intensity upon Ca2+ decrease
Source: PLoS One. 2018 Apr 25;13(4):e0194707. doi: 10.1371/journal.pone.0194707 (PMC5918796; doi:10.1371/journal.pone.0194707)
Supplement: S3 File — (PDF) [file pone.0194707.s009.pdf]

Ca2+ concentration (nM)	IP2.0	inverse-pericam
3.86	1	1
7.92	0.964337796	0.979441256
12.20	0.950457667	0.970142106
16.72	0.894356331	0.943790085
37.63	0.888083791	0.885490172
64.50	0.842818173	0.816711066
100.33	0.770177177	0.718204499
150.50	0.663180253	0.589943242
225.75	0.550431007	0.440698346
351.17	0.433577307	0.343416837
602.00	0.299315644	0.219609615
1354.50	0.199863032	0.136009707
1856.17	0.108242724	0.117339164
2859.50	0.086513995	0.100937359
5869.50	0.068455695	0.091358239
