# Supplementary material for: An improved inverse-type Ca2+ indicator can detect putative neuronal inhibition in Caenorhabditis elegans by increasing signal intensity upon Ca2+ decrease
Source: PLoS One. 2018 Apr 25;13(4):e0194707. doi: 10.1371/journal.pone.0194707 (PMC5918796; doi:10.1371/journal.pone.0194707)
Supplement: S4 File — (PDF) [file pone.0194707.s010.pdf]

IP2.0	
Initial Intensity	Intensity at first spike after histamine stimulation
1,000	0.283327709
1,491	0.235762423
1,569	0.226592938
1,587	0.236674999
1,741	0.235749302
1,836	0.229265775
2,045	0.23041249
2,237	0.19623637
2,349	0.227708523
2,395	0.231012757
2,423	0.228333438
2,437	0.236629537
2,524	0.216606801
2,633	0.24542639
2,821	0.24413042
2,917	0.240130485
2,918	0.208668711
3,153	0.209452205
3,247	0.224247832
3,250	0.208941577
3,261	0.251128716
3,287	0.22029317
3,390	0.227085638
3,400	0.219552449
3,579	0.21241826
3,770	0.165527079
3,831	0.246467137
3,923	0.206336644
4,368	0.215214982
4,784	0.216303643
4,958	0.148730498
5,102	0.204289908
5,304	0.196079784
5,322	0.191864762
5,444	0.201788131
7,472	0.204272458
7,717	0.215836657
8,748	0.222668669
9,220	0.195293958
9,817	0.195336998
10,480	0.210362828
11,790	0.185438738
20,679	0.246462888
24,243	0.162276005
