# Supplementary material for: An improved inverse-type Ca2+ indicator can detect putative neuronal inhibition in Caenorhabditis elegans by increasing signal intensity upon Ca2+ decrease
Source: PLoS One. 2018 Apr 25;13(4):e0194707. doi: 10.1371/journal.pone.0194707 (PMC5918796; doi:10.1371/journal.pone.0194707)
Supplement: S5 File — (PDF) [file pone.0194707.s011.pdf]

	IAA (%)	0.33				
	Strain		N2; Ex[Pstr-2::IP2.0]			
			qjEx15		*qjEx17*	
		N2	Ex+	Ex-	Ex+	Ex-
Plate #1	A	30	71	19	18	26
	B	5	2	0	0	2
	N	38	100	31	28	33
	chemotaxis index	0.66	0.69	0.61	0.64	0.73
Plate #2	A	56	98	54	26	12
	B	6	2	1	0	1
	N	63	161	83	37	17
	chemotaxis index	0.79	0.60	0.64	0.70	0.65
Plate #3	A	78	83	33	34	33
	B	10	1	0	2	3
	N	94	135	48	62	52
	chemotaxis index	0.72	0.61	0.69	0.52	0.58
Plate #4	A	67	35	39	40	14
	B	5	0	0	2	1
	N	75	84	59	60	23
	chemotaxis index	0.83	0.42	0.66	0.63	0.57
Plate #1-4 chemotaxis index	average	0.75	0.58	0.65	0.62	0.63
	SEM	0.04	0.06	0.02	0.04	0.04

	IAA (%)	0.1				
	Strain		N2; Ex[Pstr-2::IP2.0]			
			qjEx15		*qjEx17*	
		N2	Ex+	Ex-	Ex+	Ex-
Plate #1	A	47	73	32	25	16
	B	17	1	1	1	2
	N	69	119	53	73	33
	chemotaxis index	0.43	0.61	0.58	0.33	0.42
Plate #2	A	42	66	27	22	14
	B	15	5	0	0	0
	N	66	116	50	47	28
	chemotaxis index	0.41	0.53	0.54	0.47	0.50
Plate #3	A	38	68	32	34	13
	B	28	5	1	1	2
	N	71	119	42	74	24
	chemotaxis index	0.14	0.53	0.74	0.45	0.46
Plate #4	A	42	71	37	22	17
	B	17	6	0	0	1
	N	66	118	58	52	31
	chemotaxis index	0.38	0.55	0.64	0.42	0.52
Plate #1-4 chemotaxis index	average	0.34	0.55	0.63	0.42	0.47
	SEM	0.07	0.02	0.04	0.03	0.02

	IAA (%)	0.033				
	Strain		N2; Ex[Pstr-2::IP2.0]			
			qjEx15		*qjEx17*	
		N2	Ex+	Ex-	Ex+	Ex-
Plate #1	A	40	42	23	33	17
	B	16	3	3	4	1
	N	67	78	45	47	26
	chemotaxis index	0.36	0.50	0.44	0.62	0.62
Plate #2	A	33	38	25	30	15
	B	13	2	1	0	0
	N	54	74	42	72	25
	chemotaxis index	0.37	0.49	0.57	0.42	0.60
Plate #3	A	43	29	13	34	24
	B	9	4	4	1	1
	N	67	58	33	52	31
	chemotaxis index	0.51	0.43	0.27	0.63	0.74
Plate #4	A	43	36	22	29	23
	B	29	4	1	4	2
	N	79	55	35	60	47
	chemotaxis index	0.18	0.58	0.60	0.42	0.45
Plate #1-4 chemotaxis index	average	0.35	0.50	0.47	0.52	0.60
	SEM	0.07	0.03	0.07	0.06	0.06
